# Head Acceleration Magnitude in Sport-Related Concussive Impacts: A Systematic Review and Meta-analysis

**DOI:** 10.1007/s40279-026-02448-x

**Published:** 2026-05-26

**Authors:** Branimir Ivanic, Anna Cronström, Mana Rasi, Eva Ageberg

**Affiliations:** Department of Health Sciences, Faculty of Medicine, Lund University, Box 117, 22100 Lund, Sweden

## Abstract

**Background:**

Sport-related concussion (SRC) is a common and complex injury in athletic populations. Linear head acceleration (LHA) and rotational head acceleration (RHA) are key biomechanical factors believed to contribute to SRC, each through distinct mechanisms. Evaluating head impact magnitudes across different sports, athlete populations, and measurement methods is essential for advancing SRC injury prevention and risk assessment.

**Objective:**

We aimed to examine linear and rotational head acceleration magnitudes associated with SRC impacts in athletes participating in team sports across all ages and both sexes.

**Methods:**

We conducted a systematic review and meta-analysis adhering to the Preferred Reporting Items for Systematic reviews and Meta-Analyses (PRISMA) guidelines. We searched three databases (MEDLINE, Scopus, SPORTDiscus) until 4 September, 2024, and the literature search was updated on the 10 November, 2025. Observational and experimental studies reporting peak LHA and/or RHA during SRC impacts in team sport athletes were included. Data were extracted on study characteristics, instrumentation, and head impact magnitudes. The risk of bias was assessed using the National Institutes of Health Quality Assessment Tool, and the certainty of the evidence was evaluated using GRADE. Random-effects meta-analyses were conducted to compare SRC and non-concussive impacts, and subgroup analyses were performed by sport type, age group, sex, session type, and instrumentation type, reporting standardized mean difference and mean difference. Between-group differences were assessed using Q_b_ statistics, and heterogeneity was evaluated using the I^2^ statistics. Sensitivity and publication bias analyses were also performed.

**Results:**

Data from 30 articles representing 3262 athletes (12% female) were included. Sport-related concussion impacts produced significantly greater head acceleration magnitudes than non-concussive impacts, with large differences for both LHA (69.6 g vs 25.3 g; standardized mean difference = 2.42; 95% confidence interval 1.73–3.12) and RHA (4931 rad/s^2^ vs. 1966 rad/s^2^; standardized mean difference = 1.99; 95% confidence interval 1.17–2.81). For SRC impacts, subgroup analyses revealed significant differences across sports (*p* < 0.001), age groups (LHA only, *p* = 0.01), sexes (LHA only, *p* < 0.001), and instrumentation types (LHA *p* < 0.001; RHA *p* = 0.02). The highest LHA values were observed in American Football (83.2 g), while the highest RHA values were recorded in rugby (7627 rad/s^2^). Higher LHA values were recorded for male athletes (78.2 g) compared with female athletes (44.2 g), and for high school athletes (88.3 g) compared with youth (61.4 g) and adult athletes (72.2 g). Helmet-mounted sensors recorded the highest LHA (79.0 g), and skin patches recorded the highest RHA (6938 rad/s^2^). No significant differences were found between games and practices.

**Conclusions:**

In team sports, SRC impacts are associated with significantly higher LHA and RHA than non-concussive impacts. The observed overlap and contextual variability in head acceleration magnitudes highlight the importance of considering individual-specific and context-specific interpretation in SRC risk assessment and provide a foundation for improving head impact monitoring, injury prevention strategies, and athlete safety.

**Clinical Trial Registration:**

PROSPERO CRD42024584070.

**Supplementary Information:**

The online version contains supplementary material available at https://doi.org/10.1007/s40279-026-02448-x.

## Key Points

**Table:** 

In team sports, sport-related concussion impacts involve significantly greater linear head acceleration and rotational head acceleration than non-concussive impacts, with group-level differences averaging a 140% increase in linear head acceleration and a 135% increase in rotational head acceleration.
Substantial variability in head acceleration magnitudes was observed across subgroups, with higher values in American Football and rugby compared with other team sports, in male compared with female athletes, in high school athletes compared with youth and adults, and when using helmet-mounted sensors or skin patches compared with headband-mounted sensors.
Improving sport-related concussion research, prevention strategies, and athlete safety requires both the standardization of sensor technologies and reporting practices, and the greater inclusion of under-represented groups, particularly female athletes.

## Introduction

Sport-related concussion (SRC) is defined as: “a traumatic brain injury caused by a direct blow to the head, neck or body resulting in an impulsive force being transmitted to the brain that occurs in sports and exercise-related activities” [1]. Upon impact, the skull accelerates rapidly while the brain experiences a brief lag due to its inertia [2]. This discrepancy in movement can result in strain and pressure gradients within the brain tissue, which, if the mechanical thresholds are surpassed, have the potential to cause injury [2].

Sport-related concussion is associated with a broad range of physical (e.g., headache, dizziness), cognitive (e.g., confusion, slow processing), and/or psychological (e.g., irritability, mood changes) symptoms [3]. These symptoms may present immediately or emerge gradually, and while they typically resolve within a few days, recovery may take longer in some individuals [4]. Estimates suggest that up to 30% of affected athletes experience persistent symptoms for more than 4 weeks [5–7]. The reported increase in SRC incidence over the past decade [8–10], and the growing awareness of its potential short- and long-term health consequences [11, 12], have intensified interest in understanding the biomechanical underpinnings of head impacts. This interest has been especially pronounced in team sports, which consistently report the highest SRC incidence among both male and female athletes [9, 13] and have therefore become a central focus for understanding how linear head acceleration (LHA) and rotational head acceleration (RHA) contribute to SRC risk [14].

Given that the brain’s internal mechanical response to impact cannot be measured directly in vivo, LHA and RHA are used as key indicators of the brain’s inertial response, serving as practical surrogates for the pressure and strain experienced within neural tissues [15]. Linear head acceleration is typically associated with transient intracranial pressure changes, whereas RHA is linked to shear forces resulting from differential movement within the brain tissue [2]. Rather than acting in isolation, these accelerations often interact, with a combined effect influencing SRC’s severity and clinical outcome [16]. This interaction highlights the complex biomechanical interplay between LHA and RHA in head trauma [2, 16]. Recent technological advancements have enabled the on-field measurements of these kinematic parameters across a wide range of athlete populations and sporting environments, offering valuable insights into the biomechanics of head impacts in real-world conditions [15].

Despite significant research investments over several decades, no universally accepted biomechanical threshold can reliably distinguish SRC from non-concussive impacts [17]. Athletes frequently sustain head impacts that exceed proposed thresholds without developing a concussion, whereas some concussions occur after impacts below those levels [17]. This variability highlights the complexity of SRC biomechanics and challenges the notion of a single universal threshold for diagnosis or injury prediction across all athletes, sports, and levels of competition. Synthesizing available evidence on head acceleration magnitudes associated with SRC across different athlete groups is therefore needed to clarify how these biomechanical responses are distributed in real-world sport settings and to provide a more accurate foundation for interpreting head-impact monitoring data and developing future sport-, age-, and sex-specific risk models.

At the same time, substantial methodological variability across studies has hindered researchers’ efforts to synthesize existing knowledge [15]. Researchers use different sensor technologies, skull coupling methods, data processing algorithms, and recording thresholds (range 5–15 g or higher), influencing the impacts captured and how they are interpreted [18]. Furthermore, researchers use inconsistent concussion verification methods, as diagnostic criteria and confirmation procedures vary widely across studies [19]. Reporting practices also differ substantially across studies, including how data are processed, summarized, and stratified, which limits comparability across investigations [20]. These limitations highlight the need for a systematic and structured synthesis of the available evidence to clarify how head acceleration magnitudes differ between SRC and non-concussive impacts across study methodologies and athlete subgroups. Particular attention is warranted for female and youth athletes, who are at higher SRC risk [21, 22] yet remain under-represented in SRC and head impact research [23, 24].

To address these gaps in the current literature, this systematic review aimed to examine linear and rotational head acceleration magnitudes associated with SRC impacts in athletes participating in team sports across all ages and both sexes. By integrating available data from varied sporting contexts and measurement approaches, the review seeks to provide a more robust foundation for interpreting head impact monitoring data and support the development of future context-specific biomechanical risk models.

## Methods

### Guideline and Review Process

This review was pre-registered in PROSPERO (CRD42024584070) and was conducted in accordance with the guidelines for Preferred Reporting Items for Systematic reviews and Meta-Analyses (PRISMA) [25]. A completed PRISMA checklist is available in the Electronic Supplementary Material (ESM).

The evaluation of articles for inclusion was conducted independently by two authors (BI, MR). The extraction of the data and the assessment of the risk of bias and certainty of evidence were also conducted by these two authors. In the event of disagreement, a consensus discussion was held between the two authors, and if a consensus could not be reached, the remaining authors (AC, EA) were consulted.

### Information Sources and Search Strategy

The development of the literature search strategy was facilitated with the guidance of an experienced librarian. The search was conducted on 4 September, 2024 in the following databases: MEDLINE, Scopus, and SPORTDiscus, and was subsequently updated on 10 November, 2025. The search strategy combined subject headings (e.g., “Brain Concussion,” “Biomechanical Phenomena,” “Head Movements”) and free terms (e.g., concussion*, “head impact*,” acceleration*, linear, rotational) using Boolean operators (AND/OR) [details available in the ESM]. No language or year restrictions were applied. A citation search was conducted in the reference lists of included articles, and the systematic, scoping, and narrative reviews identified in the literature.

### Selection Process and Eligibility Criteria

All identified articles were imported into the systematic review software Covidence (2025) [26] for duplicate removal and selection process.

The inclusion criteria were: (1) full-text, original, peer-reviewed observational studies (e.g., cohort, case–control, cross-sectional, case-series studies) and experimental studies (e.g., randomized controlled trials [RCTs], cluster RCTs, and quasi-experimental studies); (2) articles including: (i) male and/or female athletes of any age, at any competitive level, participating in team sports (e.g., American Football, soccer, rugby, ice hockey); (ii) on-field match and/or training impacts that have been diagnosed as SRCs; and (iii) outcome measures of LHA and/or RHA.

The exclusion criteria were: (1) publications in languages other than English; (2) abstract-only publications and/or review articles; (3) articles including: (i) athletes participating in individual sports (e.g., boxing, mountain biking, taekwondo) and (ii) exclusively non-concussive impacts and/or impacts that occur outside of sport setting.

Sport-related concussion impacts were defined as head acceleration events associated with a SRC formally diagnosed by a certified athletic trainer, team physician, or other qualified medical professional, whereas non-concussive impacts were defined as all recorded head acceleration events that did not result in such a diagnosis. The scope of this review was restricted to team sports, as these disciplines exhibit the highest documented SRC rates across athletic populations [9, 13] and comprise the substantial majority of available head acceleration research, providing sufficient methodological consistency for meaningful synthesis and interpretation. In an effort to avoid duplication of data across articles derived from the same dataset, researchers excluded older publications when a more recent article using the same dataset was available, unless the older article contained a distinct subset of data not included in the newer publication (details available in the ESM).

### Data Collection Process

Data extraction included: (1) general study information; (2) participant number and characteristics; (3) details of the instrumentation used; (4) frequency, characteristics, and magnitude of non-concussive head impacts; and (5) frequency, characteristics, and magnitude of SRC impacts (details available in the ESM).

Age-group classification was based on the study-reported competitive or educational level, rather than chronological age alone, to better reflect differences in competitive structure and head-impact exposure. For example, youth athletes typically compete within age-restricted leagues (e.g., Bantam-level ice hockey), whereas high school athletes compete within school-based systems that include peers spanning multiple adjacent age cohorts. Consequently, athletes of similar chronological age may experience substantially different head-impact exposure depending on the competitive context.

In instances where missing data were identified, the authors were contacted via e-mail to request the necessary information. For articles that exclusively reported the magnitudes of individual SRC impacts, the mean and standard deviation (SD) were derived directly from the raw impact values, as no summary statistics were provided. The mean impact magnitude was calculated as the mean: $\overline{\mathcal{x} }=\frac{\sum {\mathcal{x}}_{\mathcal{i}}}{N}$ (${\mathcal{x}}_{\mathcal{i}}$– each individual SRC impact magnitude; N – total number of reported SRC impacts). The corresponding SD was subsequently calculated using the following formula: $\mathrm{S}\mathrm{D}= \sqrt{\frac{{\sum}_{\mathcal{i}=1}^{N}{({\mathcal{x}}_{\mathcal{i}}-\overline{\mathcal{x} })}^{2}}{N-1}}$ (${\mathcal{x}}_{\mathcal{i}}$– each individual SRC impact magnitude; $\overline{\mathcal{x} }$—mean; N—total number of reported SRC impacts). When articles reported median values, accompanied by minimum, maximum, or interquartile range measurements, the mean and SD were estimated using established statistical methods based on sample size and distributional assumptions [27]. For articles reporting only one SRC impact, the SD was imputed based on the average SD of studies with multiple SRC impacts. In instances where values of LHA or RHA were reported separately for subgroups (e.g., practice vs games, male vs female) but no overall pooled value was provided, subgroup data were combined within each study to derive an overall mean for the primary meta-analysis. When subgroup data were combined, the corresponding pooled SD was calculated using the formula: SD_mean_ = √(s_1_^2^ + s_2_^2^ + … + s_k_^2^)/k (s_*k*_—SD for *k*th group; *k*—total number of groups). For subgroup analyses, the original subgroup-specific values were retained and analyzed separately.

### Risk of Bias Assessment

The methodological quality of the included articles was assessed using the National Institutes of Health Quality Assessment Tool for Observational Cohort, Cross-Sectional, and Case-Series Studies [28]. This tool consists of 14 items that evaluate key aspects of study design, including the clarity of the research question, the appropriateness of the study population, the methods of exposure and outcome measurement, the statistical analyses, and the control of confounding variables. Each item was assigned a rating of yes, no, cannot determine, or not applicable, and the overall article quality was determined based on the number and relevance of items met.

Although the National Institutes of Health Quality Assessment Tool does not specify numerical cut-offs, general thresholds were applied to maintain consistency across evaluations. Studies meeting approximately 12–14 items were classified as good, reflecting strong methodological quality and high internal validity. Those meeting 9–11 items were rated as fair, indicating some methodological limitations but still providing relevant insights. Studies meeting eight or fewer items were rated as poor, exhibiting significant methodological weaknesses such as inadequate sample selection, insufficient control of confounders, or unreliable measurement techniques, thereby significantly reducing confidence in their findings. These thresholds were used as indicative guidelines, and the final classification also accounted for the relative importance of specific methodological aspects, particularly those influencing internal validity, measurement reliability, and bias control.

### Effect Measures and Synthesis Methods

Meta- and subgroup analyses were conducted using Stata (Version 18, 2025) [29]. The primary meta-analysis evaluated disparities in head impact magnitudes between non-concussive and SRC impacts. In a separate set of analyses, comparisons of SRC head impact magnitudes were performed to assess between-group differences in peak LHA and RHA. These subgroup analyses were conducted based on: (1) sport type—American Football, rugby, soccer, ice hockey, lacrosse, and Australian Rules Football; (2) age group—youth, high school, collegiate, and adult categories; (3) sex— male and female athletes; (4) session type—practice and games; and (5) instrumentation type—helmet-mounted sensors, skin patches, headband-mounted sensors, and instrumented mouthguards. All subgroup analyses were defined a priori, before the review was initiated.

Linear head acceleration and RHA were analyzed employing a random-effects restricted maximum likelihood method, with the outcomes presented as standardized mean differences (SMDs) and 95% confidence intervals (CIs). The interpretation of SMD effect sizes was based on conventional thresholds: small (SMD = 0.2), medium (SMD = 0.5), and large (SMD = 0.8) [30]. Because of the anticipated variability among the included articles, attributable to differences in study design, participant characteristics, and measurement methodologies, random-effect models were employed. In addition to SMDs, mean differences (MDs) were also reported to facilitate ease of interpretation.

Heterogeneity was evaluated using the I^2^ statistics, which quantify the percentage of total variability across studies attributable to heterogeneity rather than chance. The interpretation of I^2^ followed the thresholds outlined in the Cochrane Handbook for Systematic Reviews of Interventions: 0–40% (might not be important); 30–60% (may represent moderate heterogeneity); 50–90% (may represent substantial heterogeneity); 75–100% (considerable heterogeneity) [31]. The Q_b_ statistics were used as a statistical test to evaluate differences in pooled estimates between predefined subgroups.

Sensitivity analyses were conducted to examine the robustness of the findings. This was achieved by excluding articles with: (1) overall poor-quality rating; (2) head impacts not verified through video confirmation; (3) recording threshold < 10 g; and (4) single SRC impact recorded.

### Reporting Bias and Certainty Assessments

The potential for reporting bias was assessed for both LHA and RHA by visual inspection of funnel plots and statistical assessment using Egger’s test. The certainty of the evidence was assessed using the GRADEprofiler Guideline Development Tool software (GRADEpro) [32] according to the guidelines outlined in the GRADE handbook [33]. The initial quality of the evidence was rated as “low” owing to the observational nature of the included articles and could be downgraded by one or two levels based on the following five factors: (1) risk of bias; (2) imprecision; (3) inconsistency; (4) indirectness; and (5) publication bias.

## Results

### Study Selection

**Fig. 1:**
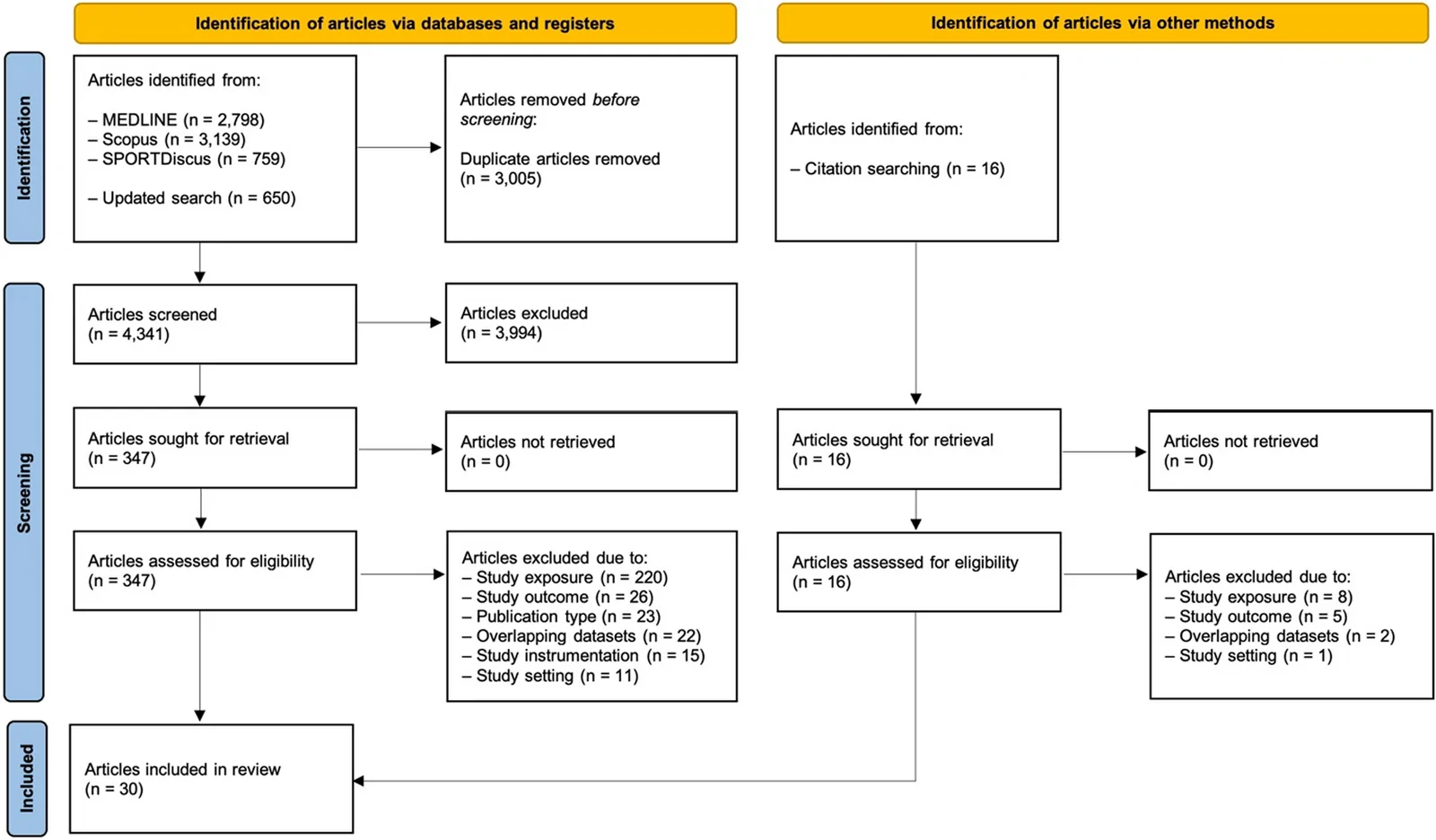
Study identification and selection Preferred Reporting Items for Systematic reviews and Meta-Analyses (PRISMA) flow diagram

The database search yielded 6696 records, with an additional 650 records identified through an updated search. In total, 347 full-text articles were screened against predefined inclusion and exclusion criteria, resulting in the exclusion of 317 articles. Of the excluded articles, 22 were excluded because of overlapping datasets with more recent publications (Fig. 1, ESM). Through the citation tracking of included articles and relevant reviews, an additional 16 articles were identified; however, none met the inclusion criteria and were therefore excluded. Consequently, 30 articles [34–63] that met all the inclusion criteria were included in the review (Fig. 1). Among the included studies, missing data were identified in two articles [37, 52], for which additional data were successfully obtained from corresponding authors and included in the meta-analysis and subgroup analyses.

### Study Characteristics

Of the 30 articles included in the review, 17 focused on athletes in American Football [34–36, 38, 40, 41, 43–45, 48, 52, 53, 56, 57, 59–61], 4 on ice hockey [50, 51, 62, 63], 3 on rugby [39, 46, 47], 2 on soccer [37, 54], and 1 on Australian Rules Football [55]. The remaining three articles examined athletes from multiple sports: two examined both lacrosse and soccer athletes [49, 58], while one focused on American Football and ice hockey athletes [42]. Regarding age groups, ten articles included youth athletes [37, 38, 40, 41, 44, 50, 51, 53, 54, 62], three included high school athletes [35, 36, 48], nine included collegiate athletes [42, 43, 45, 47, 49, 52, 58, 61, 63], and three included adult-level athletes [39, 46, 55]. Additionally, five articles included a mixed sample of high school and collegiate athletes [34, 56, 57, 59, 60]. The sample sizes ranged from four to 695 participants, with a median of 53 (interquartile range 21.5–144), while participant age ranged from a mean of 11.9 to 25.5 years across studies. In total, 88% of participants were male and 12% were female (Table 1).

**Table 1:** Characteristics of articles included in the systematic review

Study	Study design	Sport	Level	Exposure	*N*	Sex	Age (yrs)	Instr.
Beckwith, et al. 2013 [34]	Cohort	American Football	High school & Collegiate	Practice & Games	95	Male	19.2 ± 2.2	Helmet (HIT)
Broglio, et al. 2011 [35]	Cohort	American Football	High school	Practice & Games	95	Male	16.7 ± 0.8	Helmet (HIT)
Broglio, et al. 2017 [36]	Cohort	American Football	High school	Practice & Games	185	Male	16.3 ± 0.8	Helmet (HIT)
Brooks, et al. 2021 [37]	Cohort	Soccer	Youth	Games	36	Female	13.4 ± 0.9	Headband (GFT)
Campolettano,et al. 2020 [38]	Cohort	American Football	Youth	Practice & Games	15	Male	**–**	Helmet (HIT)
Carey, et al. 2019 [39]	Case series	Rugby	Adult	Games	8	Male	25.5 ± 4.7	Skinpatch (X-patch)
DiGuglielmo, et al. 2021[40]	Cohort	American Football	Youth	Games	72	Male	12.3 ± 0.5	Helmet (HIT)
Dorman, et al. 2021 [41]	Cohort	American Football	Youth	Practice & Games	103	Male	12.9 ± 0.6	Helmet (HIT)
Duhaime, et al. 2012 [42]	Cohort	American Football & Ice hockey	Collegiate	Practice & Games	450	Male & Female	**–**	Helmet (HIT)
Gabler, et al. 2020 [43]	Cohort	American Football	Collegiate	Games	21	Male	**–**	Mouthguard (Non-commercial)
Gellner, et.al. 2019 [44]	Cohort	American Football	Youth	Practice & Games	94	Male	11.9 ± 1.5	Helmet (HIT)
Hernandez et al. [45]	Cohort	American Football	Collegiate	Practice & Games	30	Male	**–**	Mouthguard (Non-commercial)
King, et al. 2015 [46]	Cohort	Rugby	Adult	Games	38	Male	22.0 ± 4.0	Mouthguard (X2)
Langevin,et al. 2021 [47]	Cohort	Rugby	Collegiate	Practice & Games	23	Female	20.2 ± 0.9	Headband (SIM)
McAlister,et al. 2023 [48]	Cohort	American Football	High school	Games	53	Male	14.4 ± 0.4	Helmet (HIT& Riddell)
Mihalik,et al. 2008 [51]	Cohort	Ice hockey	Youth	Practice & Games	14	Male	13.0	Helmet (HIT)
Mihalik et al. 2010 [50]	Cohort	Ice hockey	Youth	Games	16	Male	14.0 ± 0.5	Helmet (HIT)
Mihalik et al. 2017 [52]	Cohort	American Football	Collegiate	Practice & Games	185	Male	19.2 ± 1.4	Helmet (HIT)
Mihalik et al. 2020 [49]	Cohort	Lacrosse & Soccer	Collegiate	Practice & Games	237	Male & Female	19.8 ± 1.3	Skin patch (X-patch)
Munce et al. 2015 [53]	Cohort	American Football	Youth	Practice & Games	22	Male	12.9 ± 0.6	Helmet (HIT)
Patton, et.al. 2021 [54]	Cohort	Soccer	Youth	Games	45	Male	**–**	Headband (SIM)
Reyes, et.al. 2020 [55]	Cohort	Australian Rules Football	Adult	Games	210	Male & Female	24.3 ± 4.0	Skin patch (X-patch)
Rowson, et al. 2011 [56]	Cohort	American Football	High School & Collegiate	Practice & Games	**–**	Male	**–**	Helmet (HIT)
Rowson, et al. 2012 [57]	Cohort	American Football	High School & Collegiate	Practice & Games	335	Male	**–**	Helmet (HIT)
Sayre, et al. 2019 [58]	Case Series	Lacrosse & Soccer	Collegiate	Practice & Games	4	Female	19.2 ± 1.1	Skin patch (X-patch)
Schmidt, et al. 2014 [59]	Cohort	American Football	High School & Collegiate	Practice & Games	49	Male	17.8 ± 2.1	Helmet (HIT)
Schnebel, et al. 2007 [60]	Cohort	American Football	High School & Collegiate	Practice & Games	56	Male	**–**	Helmet (HIT)
Seifert, et al. 2022 [61]	Cohort	American Football	Collegiate	Practice & Games	695	Male	**–**	Helmet (HIT)
Swenson, et al. 2021 [62]	Cohort	Ice hockey	Youth	Practice & Games	18	Male	13.4 ± 0.7	Mouthguard (Non-commercial)
Wilcox, et al. 2015 [63]	Cohort	Ice hockey	Collegiate	Practice & Games	58	Female	**–**	Helmet (HIT)

Study	Thresh (g)	Video conf.	Head impacts	SRC	Non-Lin Acc. (g)	Non- Rot Acc. (rad/s^2^)	Con-Lin Acc. (g)	Con-Rot Acc (rad/s^2^)
Beckwith, et al. 2013 [34]	14.4	**–**	161,732	105	**–**	**–**	112.1 ± 35.4	4,253 ± 2,287
Broglio, et al. 2011 [35]	15.0	**–**	101,994	20	**–**	**–**	79.4 ± 28.3	5,563 ± 1,847
Broglio, et al. 2017 [36]	14.4	**–**	143,530	31	**–**	**–**	79.3 ± 14.3	3,401 ± 1,305
Brooks, et al. 2021 [37]	7.0	Yes	434	1	22.0 ± 8.9	**–**	45.1	**-**
Campolettano,et al. 2020 [38]	10.0	**–**	3,757	15	27.0 ± 22.9	1,296 ± 986	62.4 ± 29.7	2,609 ± 1,591
Carey, et al. 2019 [39]	10.0	Yes	2,180	6	34.2 ± 18.0	**–**	76.1 ± 17.0	**-**
DiGuglielmo, et al. 2021[40]	**–**	Yes	1,069	1	**–**	**–**	118.4	6,954
Dorman, et al. 2021 [41]	10.0	**–**	33,519	9	27.1 ±1 9.2	1,907 ± 1,380	76.2 ± 43.3	4,845 ± 2,933
Duhaime, et al. 2012 [42]	**–**	**–**	486,594	48	**–**	**–**	86.1 ± 42.6	3,620 ± 2,166
Gabler, et al. 2020 [43]	11.0, 16.0, 25.0	Yes	185	1	26.0 ± 12.0	1,900 ± 1,100	53.0	5,200
Gellner, et.al. 2019 [44]	10.0	Yes (>40)	13,909	3	**–**	**–**	59.0 ± 15.3	**–**
Hernandez et al. [45]	10.0	Yes	421	2	**–**	**–**	95.5 ± 14.8	9,970 ± 4,143
King, et al. 2015 [46]	10.0	Yes	20,687	2	22.2 ± 16.2	3,902 ± 3,948	74.9 ± 28.2	7,627 ± 3,264
Langevin,et al. 2021 [47]	15.0	Yes	120	4	30.9 ± 7.3	**–**	39.9 ± 20.7	**–**
McAlister,et al. 2023 [48]	10.0	Yes	4,678	7	29.3 ± 17.8	**–**	89.4 ± 16.9	**–**
Mihalik,et al. 2008 [51]	10.0	**–**	4,543	1	19.0 ± 14.3	**–**	86.6	**–**
Mihalik et al. 2010 [50]	10.0	Yes	666	1	21.5 ± 9.9	1,441 ± 731	31.8	2,911
Mihalik et al. 2017 [52]	10.0	Yes	283,348	24	**–**	**–**	97.6 ± 34.2	5,118 ± 3,233
Mihalik et al. 2020 [49]	10.0	**–**	35,036	9	20.5 ± 13.7	3,406 ± 3,003	72.3 ± 30.0	7,131 ± 3,613
Munce et al. 2015 [53]	10.0	**–**	6,183	1	25.5 ± 22.6	1,691 ± 1,533	52.3	4,577
Patton, et.al. 2021 [54]	16.0	Yes	30	1	42.3 ± 22.6	**–**	33.7	**–**
Reyes, et.al. 2020 [55]	10.0	Yes	4,957	2	**–**	**–**	68.5 ± 9.2	**–**
Rowson, et al. 2011 [56]	14.4	**–**	62,974	32	26.0 ± 20.0	**–**	105.0 ± 27.0	**–**
Rowson, et al. 2012 [57]	14.4	Yes	300,977	57	**–**	1,230 ± 915	**–**	5,022 ± 1,791
Sayre, et al. 2019 [58]	**–**	Yes	**–**	4	**–**	**–**	41.8 ± 29.4	6,427 ± 3,933
Schmidt, et al. 2014 [59]	10.0	**–**	19,775	1	**–**	**–**	57.7	**–**
Schnebel, et al. 2007 [60]	10.0	**–**	62,480	6	**–**	**–**	105.6 ± 34.3	**–**
Seifert, et al. 2022 [61]	10.0	No	606,943	61	**–**	**–**	59.6 ± 16.9	2,758 ± 912
Swenson, et al. 2021 [62]	5.0	Yes	892	1	11.5 ± 12.5	921.4 ± 1,049	17.3	1,677
Wilcox, et al. 2015 [63]	**–**	**–**	**–**	9	**–**	**–**	43.0 ± 11.5	4,030 ± 1,435

*Con-Lin. Acc* concussive linear acceleration, *Con-Rot. Acc.* concussive rotational acceleration, *GFT* GForce Tracker, Artaflex Inc., *HIT* Head Impact Telemetry System, Simbex, *Instr.* instrumentation, *N* sample size, *Non-Lin. Acc.* non-concussive linear acceleration, *Non-Rot. Acc.* non-concussive rotational acceleration, *Riddell* InSite Impact Response System, Riddell Inc., *SIM* Smart Impact Monitors, Triax Technologies Inc., *SRC* sport-related concussion, *Thresh.* threshold, *Video Conf.* video confirmation, *X-Patch* X2 Biosystems, *X2* X2 Biosystems, – not reported

Across the 30 included articles in the review, a total of 2,363,613 head impacts were recorded, with individual study totals that ranged from 30 to 606,943 impacts and a median of 10,046 (interquartile range 779–82,484). A total of 465 SRCs were reported across the included articles, of which 344 were explicitly associated with a single identifiable impact event. Regarding session type, 21 articles recorded head impacts during both practices and games [34–36, 38, 41, 42, 44, 45, 47, 49, 51–53, 56–63], while the remaining nine articles reported impacts from games only [37, 39, 40, 43, 46, 48, 50, 54, 55]. Instrumentation varied across articles, with 19 articles using helmet-mounted sensors [34–36, 38, 40–42, 44, 48, 50–53, 56, 57, 59–61, 63], 4 using instrumented mouthguards [43, 45, 46, 62], 4 using skin patches [39, 49, 55, 58], and 3 using headband-mounted sensors [37, 47, 54]. Recording threshold ranged from 5 to 16 g, with the most common thresholds being 10 g (used in 16 articles, [38, 39, 41, 44–46, 48–53, 55, 59–61]), followed by 14.4 g (4 articles, [34, 36, 56, 57]), 15 g (2 articles, [35, 47]), 16 g (1 article, [54]), 7 g (1 article, [37]), and 5 g (1 article, [62]). Video confirmation of SRC impacts was documented in 16 articles (53%) (Table 1).

Of the included articles, LHA values for SRC impacts were reported in 29 articles [34–56, 58–63], while RHA values were reported in 19 articles [34–36, 38, 40–43, 45, 46, 49, 50, 52, 53, 57, 58, 61–63]. A total of 16 articles reported data on non-concussive impacts, with LHA values provided in 15 [37–39, 41, 43, 46–51, 53, 54, 56, 62], and RHA values in 9 [38, 41, 43, 46, 49, 50, 53, 57, 62] (Table 1).

### Risk of Bias

Based on the overall quality assessment, no articles were rated as having good methodological quality. Of the 30 included articles, 23 (77%) were rated as having fair quality, while 7 (23%) were rated as having poor methodological quality. The majority of studies had a clearly stated research question (100%), a clearly defined study population (97%), and employed valid and reliable exposure measures (77%) and outcome measures (90%). Several common methodological limitations were identified: none of the studies provided a justification for sample size (0%), and only a few reported blinded outcome assessment (7%), follow-up rates (20%), or adjusted for potential confounding variables (3%). A detailed risk of bias assessment is available in the ESM*.*

### Synthesis of Results

#### SRC Versus Non-Concussive Head Acceleration

A total of 14 articles did not report non-concussive LHA or RHA values and were therefore excluded from the meta-analysis [34–36, 40, 42, 44, 45, 52, 55, 58–61, 63]. Consequently, the meta-analysis was conducted using 15 articles for LHA and 9 articles for RHA.

**Fig. 2:**
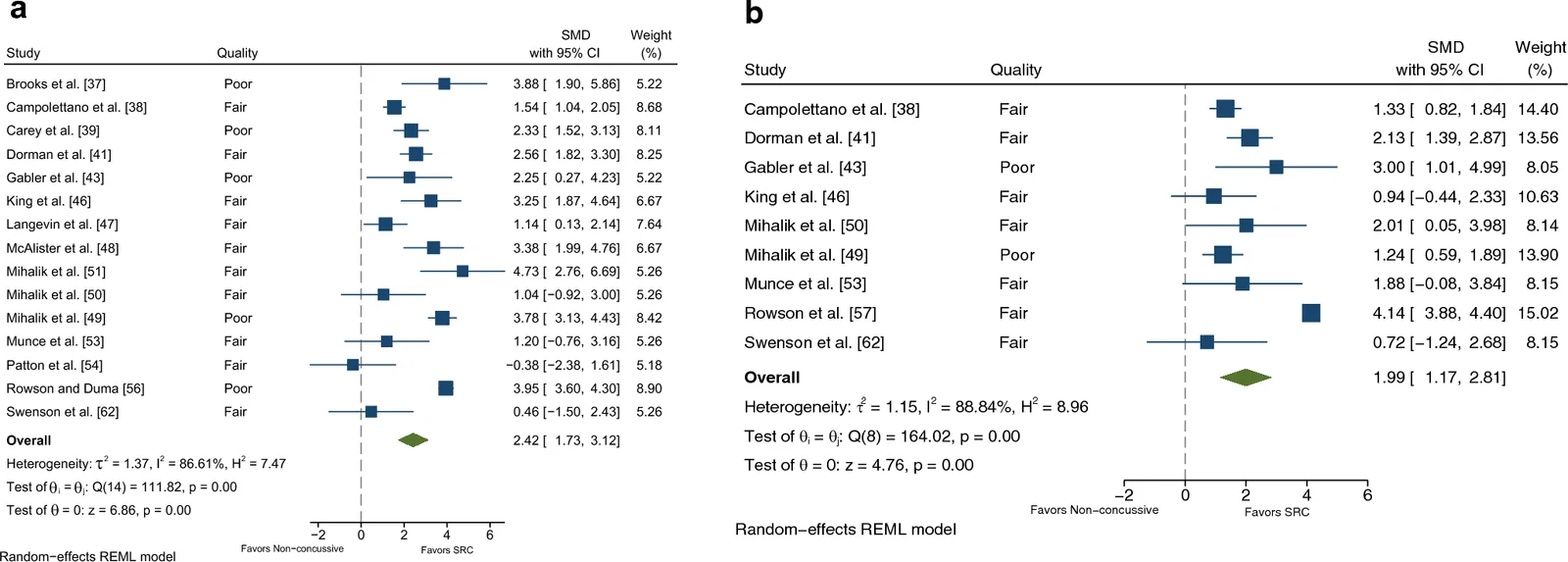
Meta-analysis of linear head acceleration (**a**) and rotational head acceleration (**b**): sport-related concussion (SRC) versus non-concussive impacts. *CI* confidence interval, *REML* restricted maximum likelihood, *SMD* standardized mean difference

For LHA, the pooled mean peak value for SRC impacts across all studies was 69.6 g, while the pooled mean for non-concussive impacts was 25.3 g, indicating large differences between the groups (SMD 2.42; 95% CI 1.73–3.12; *I*^2^ 87%; moderate-quality evidence) (Fig. 2, Table 2). On average, LHA was 37.5 g higher in SRC impacts (95% CI 25.2–49.7). For RHA, the pooled mean peak value for SRC impacts across all studies was 4931 rad/s^2^, while the pooled mean for non-concussive impacts was 1966 rad/s^2^, demonstrating large differences (SMD 1.99; 95% CI 1.17–2.81; I^2^ 89%; moderate-quality evidence) (Fig. 2, Table 2), with a mean difference of 2544 rad/s^2^ (95% CI 1673–3415).

**Table 2:** GRADE summary of findings

Outcomes	No. of head impacts (articles)	Risk of bias	Inconsistency	Indirectness	Imprecision	Publication bias	Summary of findings		Certainty
							Pooled effect	95% CI	
Linear head acceleration	175,952 (15 articles)	X	–^a^	X	X	X	SMD 2.42^b^	1.73–3.12	⨁⨁⨁◯ Moderate
Rotational head acceleration	401,996 (9 articles)	X	–^c^	X	X	X	SMD 1.99^d^	1.17–2.81	⨁⨁⨁◯ Moderate

GRADE criteria: X indicates no serious limitations; – indicates serious limitation

*CI* confidence interval, *SMD* standardized mean difference

^a^Downgraded one level because of high inconsistency (I^2^ 87%)

^b^Upgraded two levels because of large magnitude of effect (SMD 2.42)

^c^Downgraded one level because of high inconsistency (I^2^ 89%)

^d^Upgraded two levels because of large magnitude of effect (SMD 1.99)

#### Between-Group Differences in SRC Head Acceleration

##### Sport Type

**Fig. 3:**
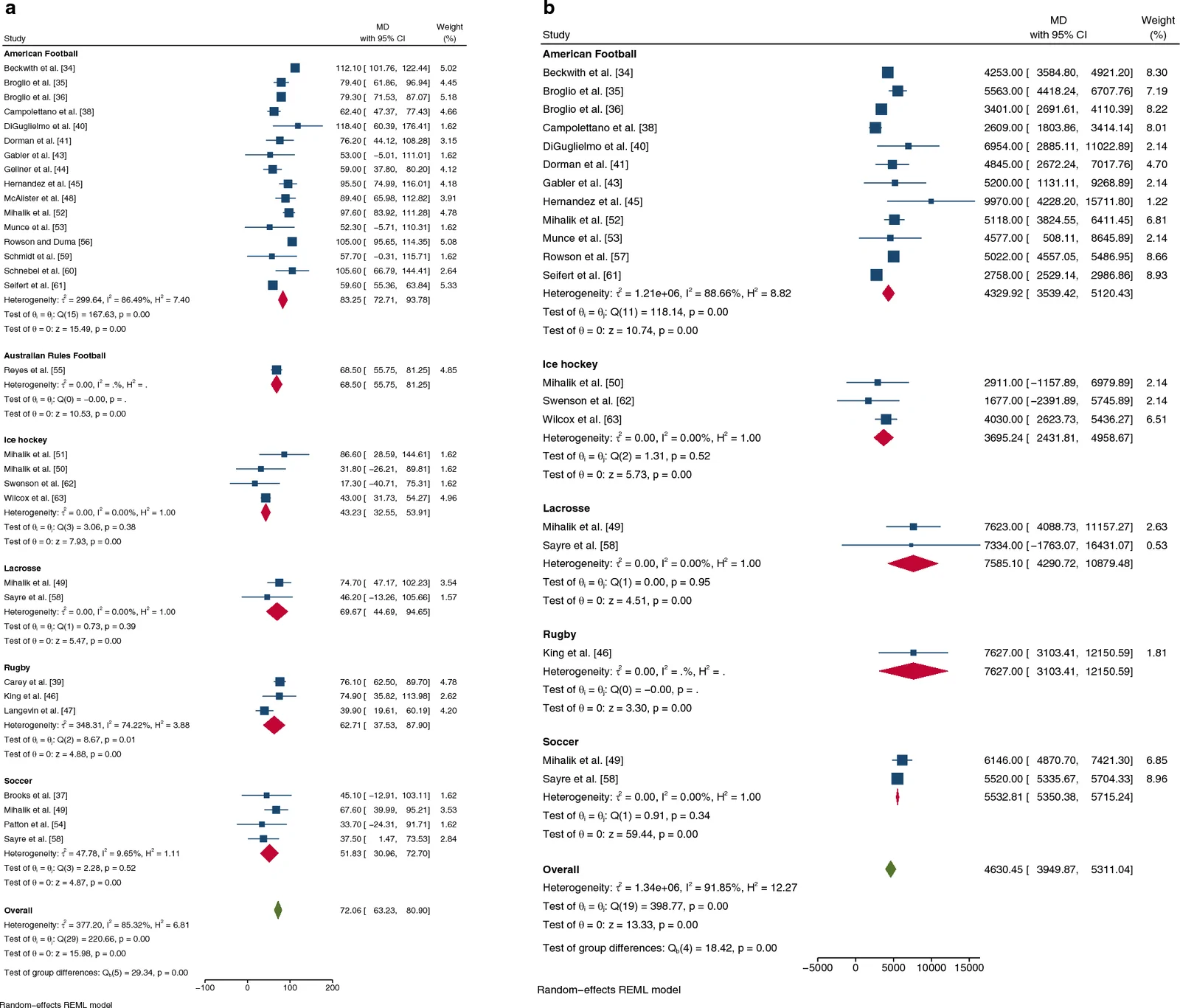
Subgroup analysis of linear head acceleration (**a**) and rotational head acceleration (**b**): sport type. *CI* confidence interval, *MD* mean difference, *REML* restricted maximum likelihood

Subgroup analyses by sport type revealed statistically significant between-group differences for both LHA (*Q*_b_ 29.3, *p* < 0.001; Fig. 3) and RHA (*Q*_b_ 18.4, *p* < 0.001; Fig. 3), indicating that the magnitude of head acceleration during SRC events varies substantially across sports. American Football recorded the highest mean peak LHA among all sports, with a pooled estimate of 83.2 g, followed by lacrosse (69.6 g), Australian Rules Football (68.5 g), rugby (62.7 g), soccer (51.8 g), and ice hockey (43.2 g). American Football had significantly higher LHA compared with ice hockey (MD = + 40.0, *p* < 0.001) and soccer (MD + 31.4 g, *p* = 0.01). Ice hockey, which showed the lowest LHA, also differed significantly from Australian Rules Football (MD = − 25.3 g, *p* < 0.001). No other between-sport comparisons in LHA reached statistical significance (ESM).

Rugby recorded the highest mean peak RHA among all sports, with a pooled estimate of 7627 rad/s^2^, followed by lacrosse (7585 rad/s^2^), soccer (5532 rad/s^2^), American Football (4329 rad/s^2^), and ice hockey (3695 rad/s^2^). Soccer had significantly higher RHA compared with American Football (MD = + 1203 rad/s^2^, *p* < 0.001) and ice hockey (MD = + 1838 rad/s^2^, *p* < 0.001). Ice hockey, which showed the lowest RHA, also differed significantly from lacrosse (MD = − 3890, *p* = 0.03). No other between-sport comparisons in RHA reached statistical significance (ESM).

##### Age Group

**Fig. 4:**
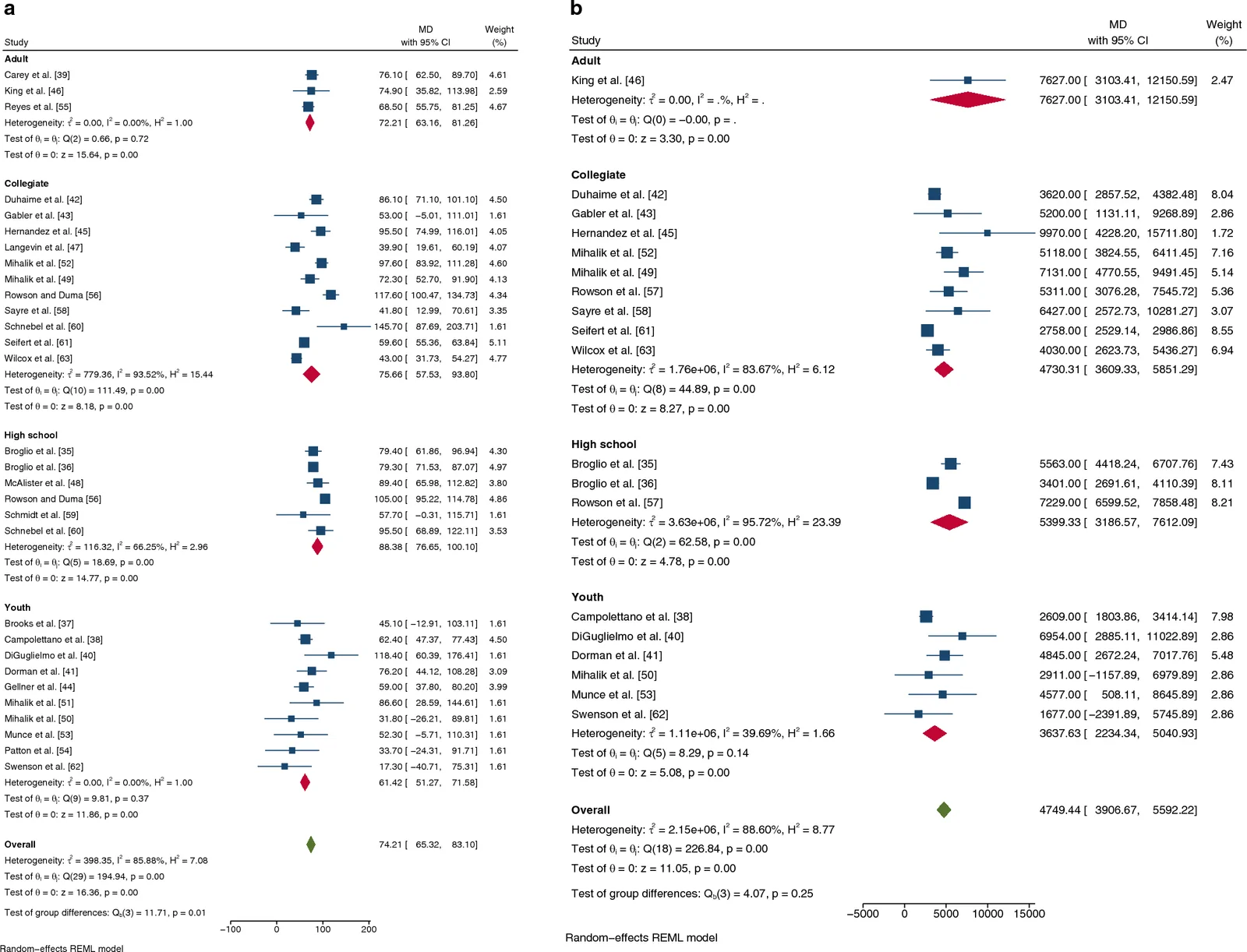
Subgroup analysis of linear head acceleration (**a**) and rotational head acceleration (**b**): age group. *CI* confidence interval, *MD* mean difference, *REML* restricted maximum likelihood

Subgroup analyses by age group revealed a statistically significant between-group difference for LHA (*Q*_b_ = 11.7, *p* = 0.01; Fig. 4), whereas no significant difference was observed for RHA (*Q*_b_ = 4.0, *p* = 0.25; Fig. 4), indicating that LHA varies across age groups, while RHA does not show statistically significant variation. High school athletes exhibited the highest mean peak LHA (88.3 g), followed by collegiate (75.6 g), adult (72.2 g), and youth (61.4 g) athletes. High school athletes had significantly higher LHA than both youth (MD = + 27.0 g, *p* < 0.001) and adult athletes (MD = + 16.2, *p* = 0.03). All other pairwise comparisons for LHA were not statistically significant (ESM).

Mean peak RHA was the highest in adults (7627 rad/s^2^), followed by high school (5399 rad/s^2^), collegiate (4730 rad/s^2^), and youth (3637 rad/s^2^) athletes. However, none of the between-group comparisons for RHA reached statistical significance (ESM).

##### Sex

**Fig. 5:**
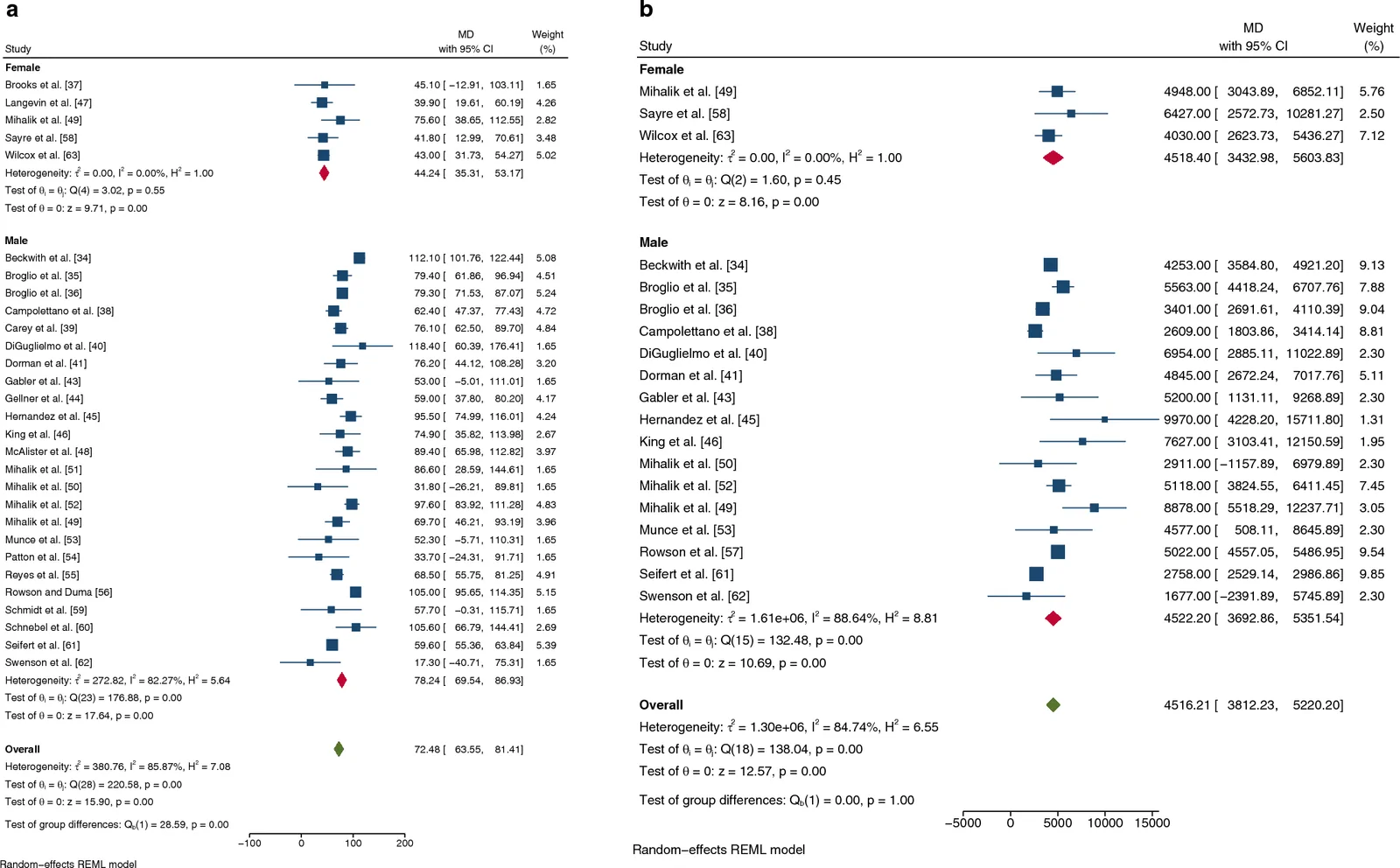
Subgroup analysis of linear head acceleration (**a**) and rotational head acceleration (**b**): sex. *CI* confidence interval, *MD* mean difference, *REML* restricted maximum likelihood

Subgroup analyses by sex revealed a statistically significant between-group difference for LHA (*Q*_b_ = 28.5, *p* < 0.001; Fig. 5), whereas no significant difference was observed for RHA (*Q*_b_ = 0.00, *p* = 1.00; Fig. 5), indicating that LHA varies across sexes, while RHA does not show a statistically significant variation. Male athletes exhibited substantially higher mean peak LHA (78.2 g), compared with female athletes (44.2 g), reflecting a mean difference of 34 g (*p* < 0.001; Fig. 5). In contrast, no significant difference was observed in RHA, with male athletes (4522 rad/s^2^) and female athletes (4518 rad/s^2^) showing nearly identical pooled estimates (*p* = 1.00; Fig. 5).

##### Session Type

**Fig. 6:**
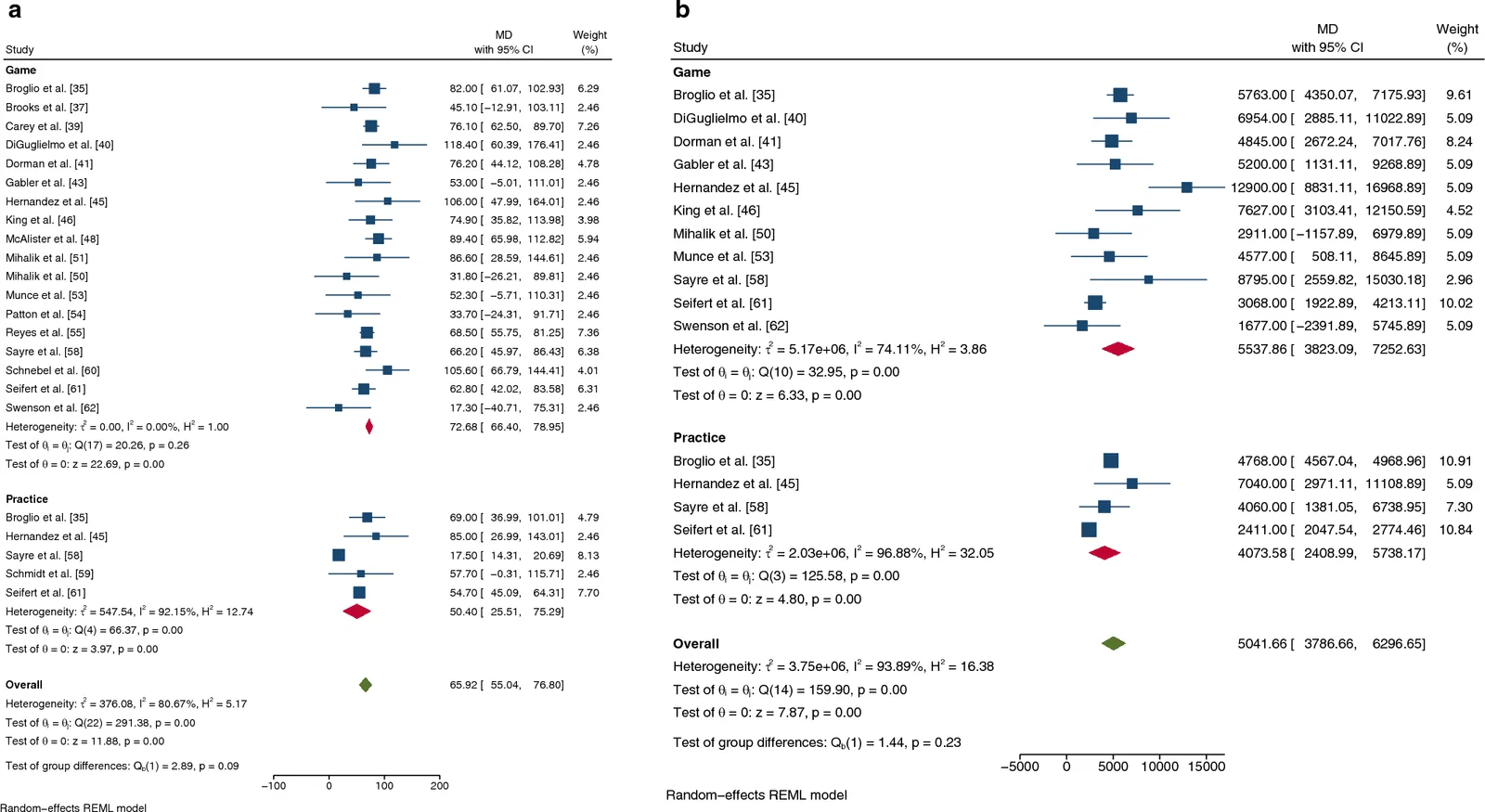
Subgroup analysis of linear head acceleration (**a**) and rotational head acceleration (**b**): session type. *CI* confidence interval, *MD* mean difference, *REML* restricted maximum likelihood

Subgroup analyses by session type revealed no statistically significant between-group differences for either LHA (*Q*_b_ = 2.89, *p* = 0.09; Fig. 6) or RHA (*Q*_b_ = 1.44, *p* = 0.23; Fig. 6), indicating that head acceleration magnitude during SRC impacts are comparable between game and practice settings. Although the difference did not reach statistical significance, both LHA and RHA values were higher during games compared with practices. Specifically, mean peak LHA was 72.6 g during games and 50.4 during practices (*p* = 0.09; Fig. 6), while mean peak RHA was 5537 rad/s^2^ during games and 4073 rad/s^2^ during practices (*p* = 0.23; Fig. 6).

##### Instrumentation Type

**Fig. 7:**
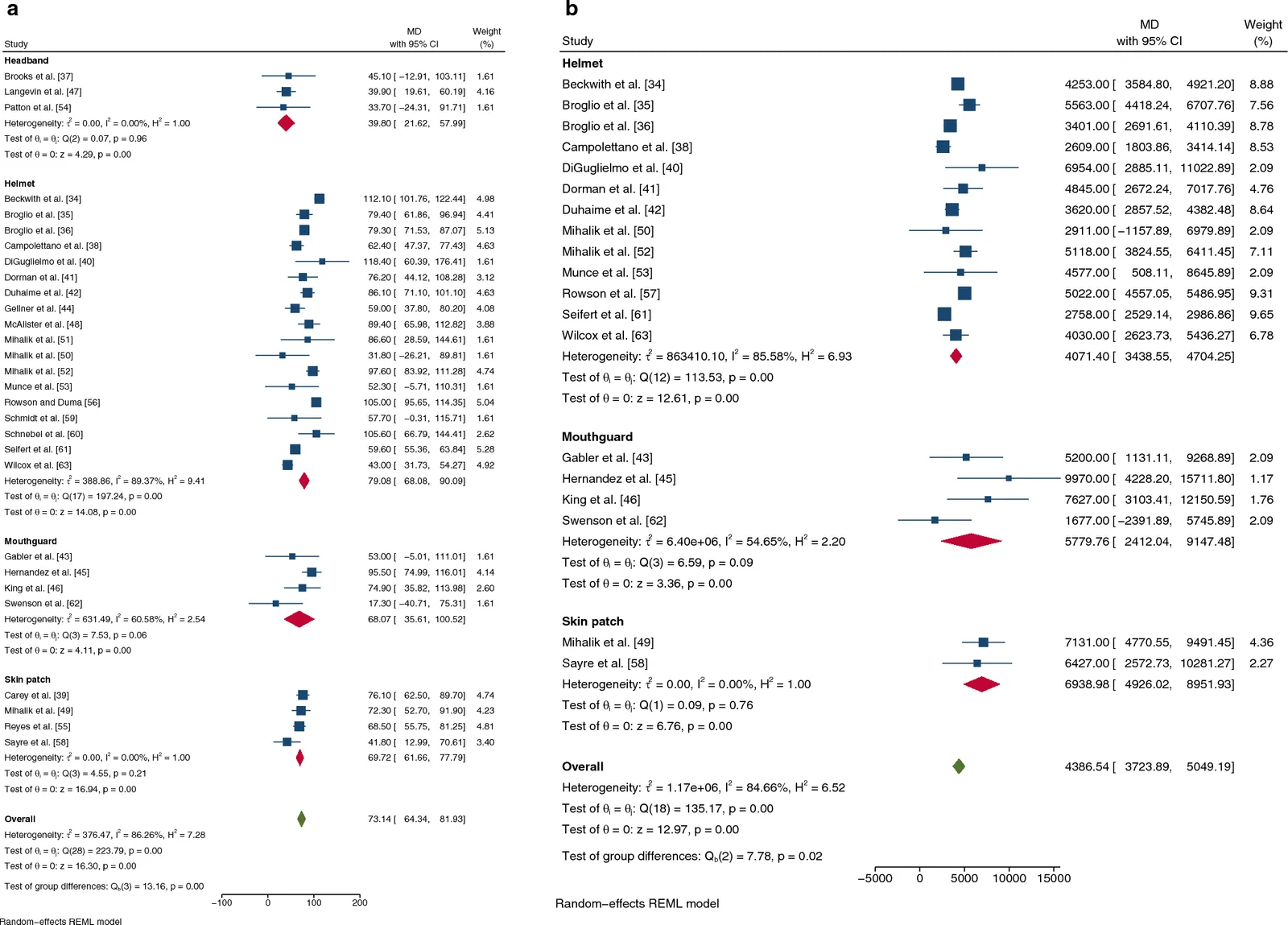
Subgroup analysis of linear head acceleration (**a**) and rotational head acceleration (**b**): instrumentation type. *CI* confidence interval, *MD* mean difference, *REML* restricted maximum likelihood

Subgroup analyses by instrumentation type revealed statistically significant between-group differences for both LHA (*Q*_b_ = 13.2, *p* < 0.001; Fig. 7) and RHA (*Q*_b_ = 7.8, *p* = 0.02; Fig. 7), indicating that the magnitude of head acceleration measured during SRC impacts differs depending on the type of sensor used. For LHA, the highest pooled mean was recorded using helmet-mounted sensors (79.0 g), followed by skin patches (69.7 g), mouthguards (68.0 g), and headbands (39.8 g). Helmets recorded significantly higher peak LHA than headbands (MD = + 39.3 g, *p* < 0.001); similarly, skin patches recorded higher values than headbands (MD = + 29.9 g, *p* < 0.001). No other pairwise comparisons reached significance (ESM).

For RHA, the highest values were reported using skin patches (6938 rad/s^2^), followed by mouthguards (5779 rad/s^2^) and helmets (4071 rad/s^2^). Skin patches recorded significantly higher values than helmets (MD = + 2868 rad/s^2^, *p* = 0.01), while all other comparisons were not statistically significant (ESM).

### Sensitivity Analyses

Sensitivity analyses demonstrated that the difference in head acceleration magnitude between SRC and non-concussive impacts remained robust across all conditions tested. For LHA, pooled SMDs ranged from 1.94 to 2.73, consistently indicating large differences between SRC and non-concussive impacts across all sensitivity analyses. Similarly, for RHA, pooled SMDs ranged from 2.00 to 2.10, likewise reflecting large and stable differences even when analyses were restricted to higher-quality studies, video-verified impacts, or alternative inclusion thresholds (ESM).

### Reporting Biases

Visual and statistical assessments were conducted for both LHA and RHA, with no evidence of potential publication bias detected (ESM).

## Discussion

This systematic review and meta-analysis included 30 articles that evaluated the magnitude of head acceleration during SRC impacts across various team sports. Sport-related concussion impacts produced head acceleration magnitudes substantially greater than those of non-concussive impacts. The certainty of evidence for this finding was rated as moderate. Subgroup analyses revealed that head acceleration magnitudes varied significantly across sports, with American Football showing the highest LHA and rugby the highest RHA; higher LHA values were also observed in male athletes compared with female athletes, and in high school athletes compared with youth and adult athletes. Measurements also differed by instrumentation type, with helmets recording the highest LHA and skin patches the highest RHA. The only subgroup comparison that did not yield a significant difference was session type (i.e., games vs practice). These findings highlight the importance of considering impact severity and contextual factors when studying the biomechanics of SRC in sports.

### SRC Versus Non-concussive Head Acceleration

The findings of this review suggest that head acceleration magnitudes are significantly greater in SRC impacts compared with non-concussive impacts. The observed differences between SRC and non-concussive impacts were statistically significant and of large magnitude, suggesting potential relevance for understanding SRC biomechanics. On average, SRC impacts involved peak LHA approximately 35.3 g higher and RHA approximately 2656 rad/s^2^ higher than non-concussive impacts. These differences correspond to a 140% increase in LHA and a 135% increase in RHA. Such magnitudes highlight the pronounced biomechanical differences between SRC and non-concussive impacts and reinforce the relevance of head acceleration as a key parameter in SRC research [64]. However, despite these group-level differences, head acceleration alone does not reliably distinguish SRC from non-concussive impacts. Previous work in American Football has shown a substantial overlap in acceleration magnitudes between SRC and non-concussive impacts [65], and a similar pattern was observed in the present review, where some SRC impacts occurred at lower magnitudes than those recorded for non-concussive impacts. This overlap suggests that although elevated acceleration increases the risk of SRC, it is insufficient as a stand-alone diagnostic marker [15]. These findings reinforce the value of acceleration metrics for characterizing the biomechanical profile of SRC, while also highlighting the need for future research to incorporate individual and contextual factors (e.g., concussion history, playing experience, positional demands) to improve interpretation and SRC risk assessment.

### Sport- and Sensor-Related Variability in Head Acceleration

Substantial variability in head acceleration magnitudes across sports and sensor types underscores the importance of contextualizing biomechanical data when studying SRC. The higher LHA observed in American Football, and the higher RHA observed in rugby, compared with the other team sports included in this review, likely result from the frequent collisions, high-intensity play, and common head impact scenarios inherent to these sports. Furthermore, in American Football, the widespread use of helmet-mounted sensors may have contributed to higher reported LHA values because of looser helmet-skull coupling [66, 67]. Under these conditions, the helmet can undergo early translational motion relative to the head, such that helmet-mounted accelerometers capture helmet inertial acceleration and shell deformation effects, inflating linear acceleration estimates compared with true head center-of-gravity motion [66, 67]. In contrast, non-helmeted sports more often use mouthguards, which typically offer closer coupling to the skull and may therefore produce more accurate estimates of head acceleration [68]. This overlap between sport type and instrumentation introduces a confounding effect that complicates direct comparisons across sports and challenges the assumption that observed differences are purely biomechanical. Moreover, variations in sensor placement, filtering algorithms, and recording thresholds further contribute to discrepancies between studies [69].

To help reduce this methodological heterogeneity, future research on head acceleration should adhere to the CHAMP 2022 Reporting Guidelines [70], which emphasize transparent reporting and consistent validation of sensor systems. Standardized disclosure of device characteristics, coupling methods, thresholds, and processing procedures would improve comparability across studies and limit the influence of instrumentation-related variability. Establishing greater methodological consistency will allow future work to more accurately identify true sport-specific, contextual, and individual differences in head acceleration, rather than differences arising from sensor or methodological inconsistency.

### Age-Related Variability in Head Acceleration

Age-related differences in head acceleration during SRC events suggest important developmental and biomechanical considerations. In this review, high school athletes exhibited the highest mean peak LHA during SRC events, significantly exceeding adult and youth athletes. This pattern may reflect the unique developmental stage of adolescence, during which rapid increases in body mass, height, and playing speed can temporarily outpace the maturation of neuromuscular control, leading to a period of transient movement instability and increased injury susceptibility [71, 72]. This differs from younger athletes, who generate lower absolute forces owing to a smaller body size and slower play, and with adults, whose cervical and neuromuscular systems are generally fully matured [73]. As cervical neuromuscular control and strength are still maturing during adolescence, high school athletes may exhibit lower cervical stabilisation capacity compared with mechanical demands of play, which can result in greater head acceleration during impacts [74] and an increased SRC risk [75, 76]. In contrast, RHA did not differ significantly across age groups. This suggests that linear forces may be more sensitive to age-related biomechanical differences or that measurement limitations obscure real differences in RHA. These findings point to a possible age-specific vulnerability, particularly for high school athletes, who appear to experience higher-magnitude linear forces during SRC events, likely reflecting the aforementioned physical and neuromuscular development. Targeted prevention strategies, such as neuromuscular warm-up programs (including neck strengthening), coaching on proper technique, and education about safe play, may be especially beneficial in this population [77]. Together with the sport- and sensor-specific differences identified in this review, these age-related patterns underscore the need for context-sensitive approaches to SRC monitoring, prevention, and management.

### Sex-Related Variability in Head Acceleration

Sex-based differences in head acceleration during SRC events were evident in this review, particularly in LHA. Male athletes exhibited substantially higher peak LHA values than female athletes, a finding that may reflect greater collision forces owing to differences in body mass, movement velocity, and intensity of contact typically seen in male-dominated sports. However, no significant difference was observed in RHA, with nearly identical pooled estimates across sexes. This could indicate that rotational accelerations during SRC events are more consistent across male and female athletes or, alternatively, that current data lack the sensitivity to detect significant sex-based differences—potentially owing to the limited number of female participants included in head impact studies. The under-representation of female athletes in SRC research remains a significant concern [78], especially given evidence suggesting that female athletes may be at greater risk for SRC and often report longer recovery times and more severe symptoms [21]. Without robust biomechanical data on SRC events in female athletes, it is difficult to determine whether existing thresholds and models adequately capture their risk profile. These findings reinforce the need for sex-specific analyses in head impact research and support greater inclusion of female athletes in future studies. Ultimately, individualized approaches to SRC monitoring and injury prevention will depend on better understanding the distinct biomechanical experiences of all athletes, including those most at risk.

### Session-Related Variability in Head Acceleration

Although head acceleration magnitudes during SRC events were not significantly different between games and practices, both LHA and RHA tended to be higher during games. These trends may reflect the increased intensity, speed, and physicality of competitive play and greater risk-taking behaviors during games [79]. However, the lack of statistical significance may reflect limited reporting of practice-related SRC events, as many studies combined game and practice data to increase sample size, thereby reducing the precision of subgroup comparisons. Notably, the presence of SRC-level impacts in both settings underscore the importance of SRC monitoring, education, and preventive strategies during practice as well as competition.

Taken together, these subgroup differences highlight important biomechanical variation across sports, age groups, sexes, instrumentation, and session types; however, their clinical relevance remains uncertain. At present, no validated injury thresholds exist, and tolerance to head acceleration likely differs across athlete populations [80]. As such, the subgroup findings presented here should be interpreted as biomechanical patterns rather than clinically relevant differences. Nonetheless, this synthesis provides population-specific acceleration data that may help inform the development of future context-specific SRC risk models.

### Head Impact Mitigation and SRC Prevention

The variability in recorded head acceleration across sports, athletes, instrumentations, and sessions also has implications for the evaluation of head impact mitigation and SRC prevention strategies. As shown in the current review, head acceleration plays a central role in SRC biomechanics, and strategies that effectively reduce the magnitude or frequency of high-risk acceleration events could have a meaningful impact on SRC risk and severity. Multiple approaches, including protective equipment (e.g., mouthguards), policy changes (e.g., disallowing bodychecking in youth ice hockey), and exercise strategies (e.g., neuromuscular warm-up programs in rugby), have demonstrated the potential to reduce SRC incidence across several team sports [77]. Additional strategies may be identified by first examining their effectiveness in reducing head impact magnitudes before progressing to injury-based evaluations. However, the interpretation of the success of these approaches depends on a clear understanding of the different exposure profiles experienced by athletes in various sporting contexts, as demonstrated in this review. Finally, greater consistency in measurement and reporting, informed by standardized approaches (e.g., CHAMP 2022 Reporting Guidelines) [70], can help ensure that the effects of mitigation strategies reflect genuine changes in SRC risk rather than differences in sport demands, athlete characteristics and history, or measurement environments.

### Strengths and Limitations

To our knowledge, this is the first systematic review and meta-analysis to comprehensively examine head acceleration magnitudes during SRC events across multiple sports, age groups, sexes, session types, and instrumentation types. By incorporating subgroup analyses and pooling data from 30 studies, this review offers novel insights into the biomechanical variability of SRC impacts and the contextual factors that may influence their measurement and interpretation. Throughout the review process, the authors diligently adhered to established guidelines to enhance transparency and the overall credibility of the systematic review.

However, several limitations should be acknowledged. First, moderate-to-substantial heterogeneity was observed in most meta- and subgroup analyses. This variability, likely driven by differences in sensor technologies, placement, data filtering, recording thresholds, and study design, also affects the interpretation of the findings by reducing comparability across studies and increasing uncertainty in the pooled estimates. In some subgroups, limited data availability and underrepresentation of certain populations — particularly female athletes — restricted the precision and generalizability of pooled estimates. Second, none of the included articles were rated as having good methodological quality, with most classified as fair. This reflects common limitations in study design and reporting, which may slightly reduce confidence in the overall findings. Third, for lacrosse, male and female athletes were pooled in the sport-level comparisons to avoid introducing sex as an additional confounder; however, this approach may have introduced bias given the known differences in protective equipment between men’s and women’s lacrosse. Fourth, while the review considered several important contextual factors, such as sex, sport, age group, session type, and instrumentation, it did not account for other potentially relevant variables, including concussion history, playing position, and athlete experience. These factors may also influence the magnitude of head acceleration during SRC events in team sports and warrant further investigation in future research.

## Conclusions

This systematic review and meta-analysis provides a comprehensive synthesis of head acceleration magnitudes during SRC events across a range of sports, athlete demographics, and sensor technologies. The findings confirm that SRC impacts are associated with significantly higher LHA and RHA than non-concussive impacts, reinforcing the central role of head acceleration in SRC biomechanics while also highlighting considerable overlap that limits its value as a stand-alone diagnostic indicator. Subgroup analyses revealed clear biomechanical variation across sports, sexes, age groups, and instrumentation types. These findings support the development of more individualized and context-specific models for SRC risk assessment and highlight the critical need for standardization in sensor technologies and reporting practices. A clearer understanding of population- and sport-specific acceleration patterns may also enhance the clinical interpretation of head impact data, supporting more informed decision making around SRC identification, monitoring, and the implementation of effective prevention strategies. Future research should prioritize the inclusion of underrepresented populations — particularly female athletes — to better inform prevention and management strategies. Collectively, these insights contribute to a more nuanced understanding of SRC biomechanics and offer a foundation for improving both on-field monitoring and long-term athlete safety.

## Supplementary Information

Below is the link to the electronic supplementary material.

Supplementary file1 (PDF 242 KB)

Supplementary file2 (PDF 164 KB)

Supplementary file3 (PDF 241 KB)

Supplementary file4 (PDF 149 KB)

Supplementary file5 (PDF 207 KB)

Supplementary file6 (PDF 137 KB)

Supplementary file7 (PDF 109 KB)

Supplementary file8 (PDF 194 KB)

## Declarations

### Availability of data and material

All data relevant to the review are included in the article or uploaded as supplemental information.
