# Supplementary material for: Head Acceleration Magnitude in Sport-Related Concussive Impacts: A Systematic Review and Meta-analysis
Source: Sports Med. 2026 May 26;56(10):2477–95. doi: 10.1007/s40279-026-02448-x (PMC13614995; doi:10.1007/s40279-026-02448-x)
Supplement: Supplementary file 2 [file 40279_2026_2448_MOESM2_ESM.pdf]

Online resource 2 – Search strategy for all databases

Head acceleration magnitude in sport-related concussive impacts: A systematic review and meta-analysis

Branimir Ivanic[1], Anna Cronström[1], Mana Rasi[1], Eva Ageberg[1]

[1]Department of Health Sciences, Faculty of Medicine, Lund University, Lund, Sweden

Journal
Sports Medicine

Corresponding author
Branimir Ivanic, Department of Health Sciences, Faculty of Medicine, Lund University, Lund, Sweden
Email: branimir.ivanic@med.lu.se
Phone: +46 739 695 279

ORCID
Branimir Ivanic: 0009-0009-5144-6516
Anna Cronström: 0000-0002-3282-6320
Mana Rasi: 0009-0002-8599-6072
Eva Ageberg: 0000-0002-8639-3006

Online resource 2 – Search strategy for all databases

Table 1. Overview of symbols and terminology.

[Mesh]<br>DE	Subject heading.
[Title/Abstract]<br>TI,AB =<br>TITLE-ABS()	Search limited to title and abstract.
*	Truncation;<br>Retrieves multiple endings for a term<br>(e.g., concussion, concussions).
OR	Boolean operator;<br>Used to combine synonyms within the same concept.
AND	Boolean operator;<br>Used to combine relevant concepts: Exposure AND Outcome.

Online resource 2 – Search strategy for all databases

Table 2. Search strategy in MEDLINE (PubMed).

Concept	#	Searches	Description
**Exposure**	1	"Brain Concussion" [Mesh]	Subject heading relevant to sport–related concussion
	2	concussion*[Title/Abstract]	Free terms relevant to sport–related concussion
	3	"mild traumatic brain injur*"[Title/Abstract]	
	4	mTBI[Title/Abstract]	
	5	"Sport-Related Concussion*"[Title/Abstract]	
	6	"Sports-Related Concussion*"[Title/Abstract]	
	7	"head impact*"[Title/Abstract]	
	8	**OR/1–7**	Combination line for Exposure concept, combined with OR
**Outcome**	9	"Head Movements" [Mesh]	Subject heading relevant to head acceleration magnitude
	10	"Biomechanical Phenomena" [Mesh]	
	11	"Acceleration" [Mesh]	
	12	kinematic*[Title/Abstract]	Free terms relevant to head acceleration magnitude
	13	acceleration*[Title/Abstract]	
	14	motion*[Title/Abstract]	
	15	magnitude[Title/Abstract]	
	16	linear[Title/Abstract]	
	17	translational[Title/Abstract]	
	18	rotational[Title/Abstract]	
	19	angular[Title/Abstract]	
	20	**OR/9–19**	Combination line for Outcome concept, combined with OR
**Search**	21	**8 AND 20**	Exposure AND Outcome

Online resource 2 – Search strategy for all databases

Table 3. Search strategy in Scopus.

Concept	#	Searches	Description
**Exposure**	1	TITLE-ABS(concussion*)	Free terms relevant to sport–related concussion
	2	TITLE-ABS("mild traumatic brain injur*")	
	3	TITLE-ABS(mTBI)	
	4	TITLE-ABS("Sport-Related Concussion*")	
	5	TITLE-ABS("Sports-Related Concussion*")	
	6	TITLE-ABS("head impact*")	
	7	**OR/1–6**	Combination line for Exposure concept, combined with OR
**Outcome**	8	TITLE-ABS(kinematic*)	Free terms relevant to head acceleration magnitude
	9	TITLE-ABS(acceleration*)	
	10	TITLE-ABS(motion*)	
	11	TITLE-ABS(magnitude)	
	12	TITLE-ABS(linear)	
	13	TITLE-ABS(translational)	
	14	TITLE-ABS(rotational)	
	15	TITLE-ABS(angular)	
	16	**OR/8–15**	Combination line for Outcome concept, combined with OR
**Search**	17	**7 AND 16**	Exposure AND Outcome

Online resource 2 – Search strategy for all databases

Table 4. Search strategy in SPORTDiscus with Full Text.

Concept	#	Searches	Description
**Exposure**	1	DE “BRAIN concussion”	Subject heading relevant to sport–related concussion
	2	TI,AB = concussion*	Free terms relevant to sport–related concussion
	3	TI,AB = “mild traumatic brain injur*”	
	4	TI,AB = mTBI	
	5	TI,AB = “Sport-Related Concussion*”	
	6	TI,AB = “Sports-Related Concussion*”	
	7	TI,AB = “head impact*”	
	8	**OR/1–7**	Combination line for Exposure concept, combined with OR
**Outcome**	9	DE “BIOMECHANICS”	Subject heading relevant to head acceleration magnitude
	10	DE “PHYSIOLOGICAL effects of acceleration”	
	11	DE “ACCELERATION (Mechanics)”	
	12	TI,AB = kinematic*	Free terms relevant to head acceleration magnitude
	13	TI,AB = acceleration*	
	14	TI,AB = motion*	
	15	TI,AB = magnitude	
	16	TI,AB = linear	
	17	TI,AB = translational	
	18	TI,AB = rotational	
	19	TI,AB = angular	
	20	**OR/9–19**	Combination line for Outcome concept, combined with OR
**Search**	21	**8 AND 20**	Exposure AND Outcome
