# Supplementary material for: Head Acceleration Magnitude in Sport-Related Concussive Impacts: A Systematic Review and Meta-analysis
Source: Sports Med. 2026 May 26;56(10):2477–95. doi: 10.1007/s40279-026-02448-x (PMC13614995; doi:10.1007/s40279-026-02448-x)
Supplement: Supplementary file 3 [file 40279_2026_2448_MOESM3_ESM.pdf]

Online resource 3 – Overlapping datasets

Head acceleration magnitude in sport-related concussive impacts:

A systematic review and meta-analysis

Branimir Ivanic[1], Anna Cronström[1], Mana Rasi[1], Eva Ageberg[1]

[1]Department of Health Sciences, Faculty of Medicine, Lund University, Lund, Sweden

Journal
Sports Medicine

Corresponding author
Branimir Ivanic, Department of Health Sciences, Faculty of Medicine, Lund University, Lund, Sweden
Email: branimir.ivanic@med.lu.se
Phone: +46 739 695 279

ORCID
Branimir Ivanic: 0009-0009-5144-6516
Anna Cronström: 0000-0002-3282-6320
Mana Rasi: 0009-0002-8599-6072
Eva Ageberg: 0000-0002-8639-3006

Online resource 3 – Overlapping datasets

Beckwith JG, et al.

Excluded Article	Included Article	Reason for Exclusion	Decision
Beckwith JG et al. 2013 (1)	Beckwith JG et al. 2013 (2)	Both articles analyzed the same dataset; however, only one clearly separated concussion with a single identified impact (n = 45).	**Included Beckwith JG, et al., 2013 (2)**<br>Excluded Beckwith JG et al., 2013 (1)

References

1. Beckwith JG, Greenwald RM, Chu JJ, Crisco JJ, Rowson S, Duma SM, Broglio SP, McAllister TW, Guskiewicz KM, Mihalik JP, Anderson S, Schnebel B, Brolinson PG, Collins MW. Timing of concussion diagnosis is related to head impact exposure prior to injury. Med Sci Sports Exerc. 2013 Apr;45(4):747-54.
2. Beckwith JG, Greenwald RM, Chu JJ, Crisco JJ, Rowson S, Duma SM, Broglio SP, McAllister TW, Guskiewicz KM, Mihalik JP, Anderson S, Schnebel B, Brolinson PG, Collins MW. Head impact exposure sustained by football players on days of diagnosed concussion. Med Sci Sports Exerc. 2013 Apr;45(4):737-46.

Online resource 3 – Overlapping datasets

Campolettano ET, et al.

Excluded Article	Included Article	Reason for Exclusion	Decision
Cobb BR, et al. 2013	Campolettano ET, et al. 2020	Single concussion case included in broader dataset reported by Campolettano ET, et al. 2020	**Included Campolettano ET, et al. 2020**<br>Excluded Cobb BR, et al. 2013<br>Excluded Daniel RW, et al. 2014<br>Excluded Campolettano ET, et al. 2018
Daniel RW, et al. 2014		Three concussion cases included in broader dataset reported by Campolettano ET, et al. 2020	
Campolettano ET, et al. 2018		Single concussion case included in broader dataset reported by Campolettano ET, et al. 2020	

References

1. Cobb BR, Urban JE, Davenport EM, Rowson S, Duma SM, Maldjian JA, Whitlow CT, Powers AK, Stitzel JD. Head impact exposure in youth football: elementary school ages 9-12 years and the effect of practice structure. Ann Biomed Eng. 2013 Dec;41(12):2463-73.
2. Daniel RW, Rowson S, Duma SM. Head impact exposure in youth football: middle school ages 12-14 years. J Biomech Eng. 2014 Sep;136(9):094501.
3. Campolettano ET, Gellner RA, Rowson S. Relationship between Impact Velocity and Resulting Head Accelerations during Head Impacts in Youth Football. Proc Int IRCOBI Conf Biomech Impacts. 2018 Sep;2018:326-333.
4. Campolettano ET, Gellner RA, Smith EP, Bellamkonda S, Tierney CT, Crisco JJ, Jones DA, Kelley ME, Urban JE, Stitzel JD, Genemaras A, Beckwith JG, Greenwald RM, Maerlender AC, Brolinson PG, Duma SM, Rowson S. Development of a Concussion Risk Function for a Youth Population Using Head Linear and Rotational Acceleration. Ann Biomed Eng. 2020 Jan;48(1):92-103.

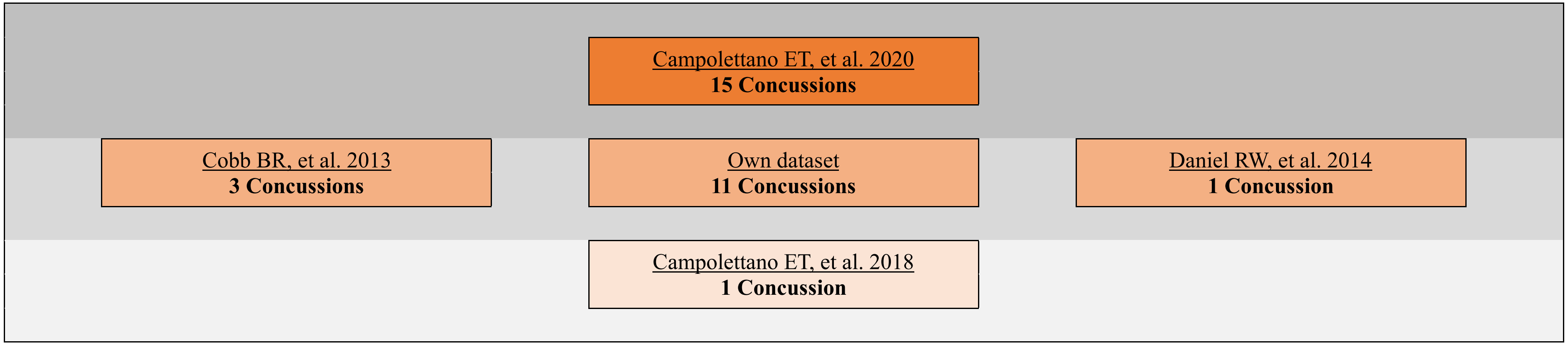

Online resource 3 – Overlapping datasets

Care Consortium

<table>
<tr><th>Excluded Article</th><th>Included Article</th><th>Reason for Exclusion</th><th>Decision</th></tr>
<tr><td>Rowson S, et al. 2018</td><td rowspan="9">Seifert J, et al. 2022</td><td rowspan="9">All articles used the CARE Consortium dataset. The included article was the most recent and comprehensive version. Earlier articles excluded to avoid overlapping datasets.</td><td rowspan="9">Included Seifert J, et al. 2022
Excluded Rowson S, et al. 2018
Excluded Rowson S, et al. 2019
Excluded Stemper BD, et al. 2019 (1)
Excluded Stemper BD, et al. 2019 (2)
Excluded Bartsch AJ, et al. 2020
Excluded Mihalik JP, et al. 2020
Excluded Rowson S, et al. 2020
Excluded McCrea MA, et al. 2021
Excluded Stemper, et al. 2022</td></tr>
<tr><td>Rowson S, et al. 2019</td></tr>
<tr><td>Stemper BD, et al. 2019 (1)</td></tr>
<tr><td>Stemper BD, et al. 2019 (2)</td></tr>
<tr><td>Bartsch AJ, et al. 2020</td></tr>
<tr><td>Mihalik JP, et al. 2020</td></tr>
<tr><td>Rowson S, et al. 2020</td></tr>
<tr><td>McCrea MA, et al. 2021</td></tr>
<tr><td>Stemper, et al. 2022</td></tr>
</table>

References

1. Rowson S, Duma SM, Stemper BD, Shah A, Mihalik JP, et al. Correlation of Concussion Symptom Profile with Head Impact Biomechanics: A Case for Individual-Specific Injury Tolerance. J Neurotrauma. 2018 Feb 15;35(4):681-690.
2. Rowson S, Campolettano ET, Duma SM, Stemper B, Shah A, et al. Accounting for Variance in Concussion Tolerance Between Individuals: Comparing Head Accelerations Between Concussed and Physically Matched Control Subjects. Ann Biomed Eng. 2019 Oct;47(10):2048-2056.
3. Stemper BD, Shah AS, Harezlak J, Rowson S, Mihalik JP, et al. Comparison of Head Impact Exposure Between Concussed Football Athletes and Matched Controls: Evidence for a Possible Second Mechanism of Sport-Related Concussion. Ann Biomed Eng. 2019 Oct;47(10):2057-2072.
4. Stemper BD, Shah AS, Harezlak J, Rowson S, Duma S, et al. Repetitive Head Impact Exposure in College Football Following an NCAA Rule Change to Eliminate Two-A-Day Preseason Practices: A Study from the NCAA-DoD CARE Consortium. Ann Biomed Eng. 2019 Oct;47(10):2073-2085.
5. Bartsch AJ, Hedin D, Alberts J, Benzel EC, Cruickshank J, et al. High Energy Side and Rear American Football Head Impacts Cause Obvious Performance Decrement on Video. Ann Biomed Eng. 2020 Nov;48(11):2667-2677.
6. Mihalik JP, Chandran A, Powell JR, Roby PR, Guskiewicz KM, et al. Do Head Injury Biomechanics Predict Concussion Clinical Recovery in College American Football Players? Ann Biomed Eng. 2020 Nov;48(11):2555-2565.
7. Rowson S, Campolettano ET, Duma SM, Stemper B, Shah A, et al. Concussion Risk Between Individual Football Players: Survival Analysis of Recurrent Events and Non-events. Ann Biomed Eng. 2020 Nov;48(11):2626-2638.
8. McCrea MA, Shah A, Duma S, Rowson S, Harezlak J, et al. Opportunities for Prevention of Concussion and Repetitive Head Impact Exposure in College Football Players: A Concussion Assessment, Research, and Education (CARE) Consortium Study. JAMA Neurol. 2021 Mar 1;78(3):346-350.
9. Stemper BD, Harezlak J, Shah AS, Rowson S, Mihalik JP, et al. Association between Preseason/Regular Season Head Impact Exposure and Concussion Incidence in NCAA Football. Med Sci Sports Exerc. 2022 Jun 1;54(6):912-922.
10. Seifert J, Shah AS, Harezlak J, Rowson S, Mihalik JP, et al. Time Delta Head Impact Frequency: An Analysis on Head Impact Exposure in the Lead Up to a Concussion: Findings from the NCAA-DOD Care Consortium. Ann Biomed Eng. 2022 Nov;50(11):1473-1487.

Online resource 3 – Overlapping datasets

Mihalik JP, et al.

Excluded Article	Included Article	Reason for Exclusion	Decision
Liao S, et al. 2016	Mihalik JP, et al. 2017	Both articles used the identical dataset. The included article was the most recent and comprehensive version. Earlier article excluded to avoid overlapping datasets.	**Included Mihalik JP, et al. 2017**<br>Excluded Liao S, et al. 2016

References

1. Liao S, Lynall RC, Mihalik JP. The Effect of Head Impact Location on Day of Diagnosed Concussion in College Football. Med Sci Sports Exerc. 2016 Jul;48(7):1239-43.
2. Mihalik JP, Lynall RC, Wasserman EB, Guskiewicz KM, Marshall SW. Evaluating the "Threshold Theory": Can Head Impact Indicators Help? Med Sci Sports Exerc. 2017 Feb;49(2):247-253. doi: 10.1249/MSS.0000000000001089. Erratum in: Med Sci Sports Exerc. 2019 Mar;51(3):605.

Online resource 3 – Overlapping datasets

Virginia Tech (Linear Head Acceleration)

<table>
<tr><th>Excluded Article</th><th>Included Article</th><th>Reason for Exclusion</th><th>Decision</th></tr>
<tr><td>Duma SM, et al. 2005</td><td rowspan="6">Rowson S, et al. 2011</td><td rowspan="6">These articles share overlapping datasets. Each successive study included data from earlier publications, with small additions over time. To avoid duplication and maximize completeness, most recent article was retained.</td><td rowspan="6">Included Rowson S, et al. 2011
Excluded Duma SM, et al. 2005
Excluded Brolinson PG, et al. 2006
Excluded Funk JR, 2007
Excluded Guskiewicz KM, et al. 2007
Excluded Duma SM, et al. 2009
Excluded Broglio SP, et al. 2010</td></tr>
<tr><td>Brolinson PG, et al. 2006</td></tr>
<tr><td>Funk JR, 2007</td></tr>
<tr><td>Guskiewicz KM, et al. 2007</td></tr>
<tr><td>Duma SM, et al. 2009</td></tr>
<tr><td>Broglio SP, et al. 2010</td></tr>
</table>

References

1. Duma SM, et al. Analysis of real-time head accelerations in collegiate football players. Clin J Sport Med. 2005 Jan;15(1):3-8.
2. Brolinson PG, Manoogian S, McNeely D, Goforth M, Greenwald R, Duma S. Analysis of linear head accelerations from collegiate football impacts. Curr Sports Med Rep. 2006 Feb;5(1):23-8.
3. Funk JR, Duma SM, Manoogian SJ, Rowson S. Biomechanical risk estimates for mild traumatic brain injury. Annu Proc Assoc Adv Automot Med. 2007;51:343-61.
4. Guskiewicz KM, et al. Measurement of head impacts in collegiate football players: relationship between head impact biomechanics and acute clinical outcome after concussion. Neurosurgery. 2007 Dec;61(6):1244-52
5. Duma SM, Rowson S. Every Newton Hertz: a macro to micro approach to investigating brain injury. Annu Int Conf IEEE Eng Med Biol Soc. 2009;2009:1123-6.
6. Broglio SP, Schnebel B, Sosnoff JJ, Shin S, Fend X, He X, Zimmerman J. Biomechanical properties of concussions in high school football. Med Sci Sports Exerc. 2010 Nov;42(11):2064-71.
7. Rowson S, Duma SM. Development of the STAR evaluation system for football helmets: integrating player head impact exposure and risk of concussion. Ann Biomed Eng. 2011 Aug;39(8):2130-40.

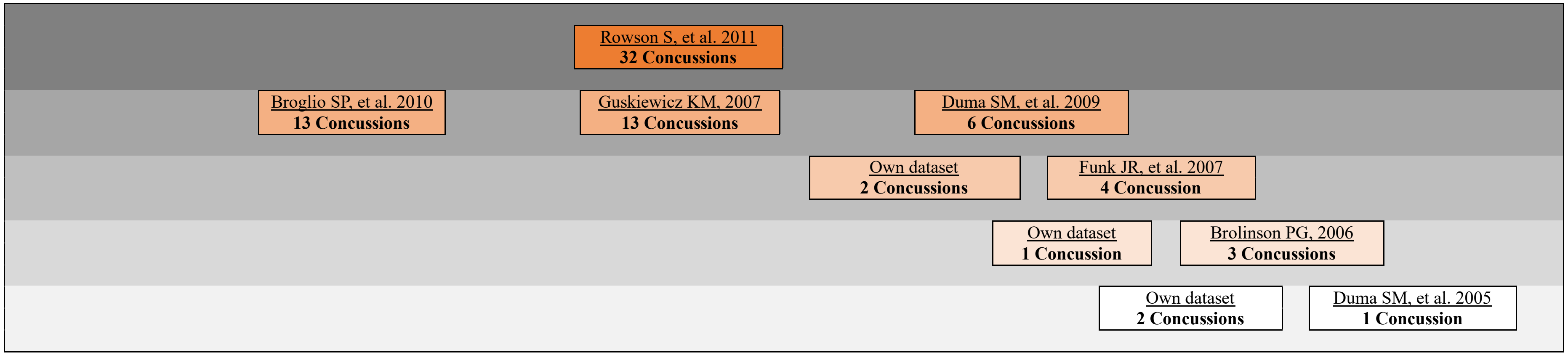

Online resource 3 – Overlapping datasets

Virginia Tech (Rotational Head Acceleration)

Excluded Article	Included Article	Reason for Exclusion	Decision
Guskiewicz KM, et al. 2007	Rowson S, et al. 2012	13 concussion cases included in broader dataset reported by Rowson S, et al. 2012	**Included Rowson S, et al. 2012**<br>Excluded Guskiewicz KM, et al. 2007<br>Excluded Broglio SP, et al. 2010
Broglio SP, et al. 2010		13 concussion cases included in broader dataset reported by Rowson S, et al. 2012	

References

1. Guskiewicz KM, Mihalik JP, Shankar V, Marshall SW, Crowell DH, et al. Measurement of head impacts in collegiate football players: relationship between head impact biomechanics and acute clinical outcome after concussion. Neurosurgery. 2007 Dec;61(6):1244-52
2. Broglio SP, Schnebel B, Sosnoff JJ, Shin S, Fend X, He X, Zimmerman J. Biomechanical properties of concussions in high school football. Med Sci Sports Exerc. 2010 Nov;42(11):2064-71.
3. Rowson S, Duma SM, Beckwith JG, Chu JJ, Greenwald RM, Crisco JJ, Brolinson PG, Duhaime AC, McAllister TW, Maerlender AC. Rotational head kinematics in football impacts: an injury risk function for concussion. Ann Biomed Eng. 2012 Jan;40(1):1-13.

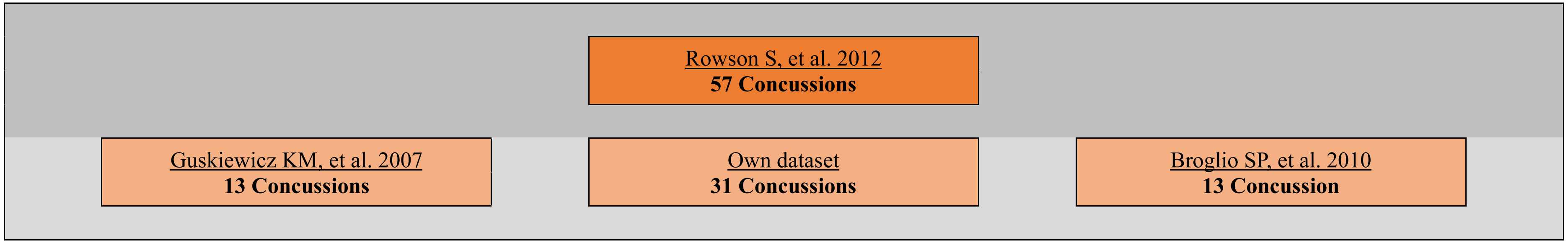

Online resource 3 – Overlapping datasets

Broglio SP, et al.

Excluded Article	Included Article	Reason for Exclusion	Decision
Broglio SP, et al. 2010	Rowson S, et al. 2011<br>Rowson S, et al. 2012	13 concussion cases included in broader dataset reported by Rowson S, et al. 2011 & Rowson S, et al. 2012.	**Include Rowson S, et al. 2011**<br>**Include Rowson S, et al. 2012**<br>Exclude Broglio SP, et al. 2010
10 out of 20 concussions excluded in Broglio SP, et al. 2011	10/20 concussions included in Broglio SP, et al. 2011	Dataset overlap between Broglio SP, et al. 2010 and Broglio SP, et al. 2011.	**10 out of 20 concussions** from Broglio SP, et al. 2011 were included.
Eckner JT, et al. 2011	Broglio SP, et al. 2011	Identical dataset to Broglio SP, et al. 2011	**Include Broglio SP, et al. 2011**<br>Exclude Eckner JT, et al. 2011
13 out of 31 concussions excluded in Broglio SP, et al. 2017	13/31 concussions included in Broglio SP, et al. 2017	Dataset overlap between Broglio SP, et al. 2010, Broglio SP, et al. 2011, and Broglio SP, et al. 2017.	**13 out of 31 concussions** from Broglio SP, et al. 2017 were included.

References

1. Broglio SP, Schnebel B, Sosnoff JJ, Shin S, Fend X, He X, Zimmerman J. Biomechanical properties of concussions in high school football. Med Sci Sports Exerc. 2010 Nov;42(11):2064-71.
2. Eckner JT, Sabin M, Kutcher JS, Broglio SP. No evidence for a cumulative impact effect on concussion injury threshold. J Neurotrauma. 2011 Oct;28(10):2079-90.
3. Broglio SP, Eckner JT, Surma T, Kutcher JS. Post-concussion cognitive declines and symptomatology are not related to concussion biomechanics in high school football players. J Neurotrauma. 2011 Oct;28(10):2061-8.
4. Broglio SP, Lapointe A, O'Connor KL, McCrea M. Head Impact Density: A Model To Explain the Elusive Concussion Threshold. J Neurotrauma. 2017 Oct 1;34(19):2675-2683.
5. Rowson S, Duma SM. Development of the STAR evaluation system for football helmets: integrating player head impact exposure and risk of concussion. Ann Biomed Eng. 2011 Aug;39(8):2130-40.
6. Rowson S, Duma SM, Beckwith JG, Chu JJ, Greenwald RM, Crisco JJ, Brolinson PG, Duhaime AC, McAllister TW, Maerlender AC. Rotational head kinematics in football impacts: an injury risk function for concussion. Ann Biomed Eng. 2012 Jan;40(1):1-13.
