# Supplementary material for: Head Acceleration Magnitude in Sport-Related Concussive Impacts: A Systematic Review and Meta-analysis
Source: Sports Med. 2026 May 26;56(10):2477–95. doi: 10.1007/s40279-026-02448-x (PMC13614995; doi:10.1007/s40279-026-02448-x)
Supplement: Supplementary file 4 [file 40279_2026_2448_MOESM4_ESM.pdf]

Online resource 4 – Extracted data items

Head acceleration magnitude in sport-related concussive impacts: A systematic review and meta-analysis

Branimir Ivanic[1], Anna Cronström[1], Mana Rasi[1], Eva Ageberg[1]

[1]Department of Health Sciences, Faculty of Medicine, Lund University, Lund, Sweden

Journal
Sports Medicine

Corresponding author
Branimir Ivanic, Department of Health Sciences, Faculty of Medicine, Lund University, Lund, Sweden
Email: branimir.ivanic@med.lu.se
Phone: +46 739 695 279

ORCID
Branimir Ivanic: 0009-0009-5144-6516
Anna Cronström: 0000-0002-3282-6320
Mana Rasi: 0009-0002-8599-6072
Eva Ageberg: 0000-0002-8639-3006

Online resource 4 – Extracted data items

General study information

- Author
- Study title
- Publication year
- Study design
- Study objectives

Characteristics of study participants

- Number of participants
- Sport (e.g., American Football, rugby, soccer)
- Age
- Sex
- Competitive level (e.g., youth, high school, collegiate)

Exposure characteristics

- Type of exposure (e.g., practice, game)
- Number of head impacts
- Number of concussive events
- Instrumentation used (e.g., instrumented helmets, skin patch, instrumented mouthguards)
- Recording threshold (e.g., 7g, 10g, 14.4g)
- Video confirmation

Outcomes measured

- Linear head acceleration
- Rotational head acceleration

Data extracted

1) Linear head acceleration
   - Mean/median values, standard deviation (SD)/interquartile range (IQR), number of head impacts in concussive/non-concussive group

2) Rotational head acceleration
   - Mean/median values, standard deviation (SD)/interquartile range (IQR), number of head impacts in concussive/non-concussive group
