# Supplementary material for: Head Acceleration Magnitude in Sport-Related Concussive Impacts: A Systematic Review and Meta-analysis
Source: Sports Med. 2026 May 26;56(10):2477–95. doi: 10.1007/s40279-026-02448-x (PMC13614995; doi:10.1007/s40279-026-02448-x)
Supplement: Supplementary file 5 [file 40279_2026_2448_MOESM5_ESM.pdf]

Online resource 5 – Risk of bias assessment

Head acceleration magnitude in sport-related concussive impacts: A systematic review and meta-analysis

Branimir Ivanic[1], Anna Cronström[1], Mana Rasi[1], Eva Ageberg[1]

[1]Department of Health Sciences, Faculty of Medicine, Lund University, Lund, Sweden

Journal
Sports Medicine

Corresponding author
Branimir Ivanic, Department of Health Sciences, Faculty of Medicine, Lund University, Lund, Sweden
Email: branimir.ivanic@med.lu.se
Phone: +46 739 695 279

ORCID
Branimir Ivanic: 0009-0009-5144-6516
Anna Cronström: 0000-0002-3282-6320
Mana Rasi: 0009-0002-8599-6072
Eva Ageberg: 0000-0002-8639-3006

Online resource 5 – Risk of bias assessment

Table 1. Risk of bias assessment (NIH Quality Assessment Tool).

Author and publication year	1. Clearly Stated Research Question	2. Defined Study Population	3. Participation Rate ≥ 50%	4. Consistent Recruitment Criteria	5. Sample Size Justification	6. Exposure Measured Before Outcome	7. Sufficient Timeframe for Outcomes	8. Different Exposure Levels Examined	9. Valid and Reliable Exposure Measures	10. Repeated Exposure Assessments	11. Valid and Reliable Outcome Measures	12. Blinded Outcome Assessment	13. Acceptable Follow-up Rate (≤ 20% Loss)	14. Adjustment for Confounding Variables		**Total Score**	**Overall Quality Rating**
**Beckwith, et al. 2013**	+	+	?	+	–	+	+	+	+	+	+	?	?	–		9	Fair
**Broglio, et al. 2011**	+	+	?	+	–	+	+	+	+	+	+	?	?	–		9	Fair
**Broglio, et al. 2017**	+	+	?	+	–	+	+	+	+	+	+	?	?	–		9	Fair
**Brooks, et al. 2021**	+	+	?	+	–	+	+	+	–	+	?	?	?	–		7	Poor
**Campolettano, et al. 2020**	+	+	+	+	–	+	+	+	+	+	+	?	?	–		10	Fair
**Carey, et al. 2019**	+	+	–	+	–	+	+	+	?	+	?	?	+	–		8	Poor
**DiGuglielmo, et al. 2021**	+	+	?	+	–	+	+	+	+	+	+	?	?	–		9	Fair
**Dorman, et al. 2021**	+	+	+	+	–	+	+	+	+	+	+	?	+	–		11	Fair
**Duhaime, et al. 2012**	+	+	?	+	–	+	+	+	+	+	+	?	?	–		9	Fair
**Gabler, et al. 2020**	+	+	?	+	–	+	–	+	+	+	+	?	?	–		8	Poor

Online resource 5 – Risk of bias assessment

**Gellner, et al. 2019**	+	+	?	+	–	+	+	+	+	+	+	?	?	–		9	Fair
**Hernandez, et al. 2015**	+	+	–	+	–	+	+	+	+	+	+	?	?	–		9	Fair
**King, et al. 2015**	+	+	+	+	–	+	+	+	+	+	+	?	?	–		10	Fair
**Langevin, et al. 2021**	+	+	+	+	–	+	+	+	+	+	+	?	?	–		10	Fair
**McAlister, et al. 2023**	+	+	?	+	–	+	+	+	+	+	+	?	–	+		10	Fair
**Mihalik, et al. 2008**	+	+	?	+	–	+	+	+	+	+	+	?	?	–		9	Fair
**Mihalik, et al. 2010**	+	+	?	+	–	+	+	+	+	+	+	?	?	–		9	Fair
**Mihalik, et al. 2017**	+	+	?	+	–	+	+	+	?	+	+	+	+	–		10	Fair
**Mihalik, et al. 2020**	+	+	+	+	–	+	+	+	?	+	?	?	?	–		8	Poor
**Munce, et al. 2015**	+	+	?	+	–	+	+	+	+	+	+	?	+	–		10	Fair
**Patton, et al. 2021**	+	+	+	+	–	+	+	+	?	+	+	?	?	–		9	Fair
**Reyes, et al. 2020**	+	+	+	+	–	+	+	+	?	–	+	?	?	–		8	Poor
**Rowson, et al. 2011**	+	–	–	+	–	+	+	+	+	?	+	?	?	–		7	Poor
**Rowson, et al. 2012**	+	+	?	+	–	+	+	+	+	+	+	?	?	–		9	Fair
**Sayre, et al. 2019**	+	+	?	+	–	+	+	+	?	+	+	?	+	–		9	Poor
**Schmidt, et al. 2014**	+	+	+	+	–	+	+	+	+	+	+	?	?	–		10	Fair
**Schnebel, et al. 2007**	+	+	?	+	–	+	+	+	+	+	+	?	?	–		9	Fair
**Seifert, et al. 2022**	+	+	?	+	–	+	+	+	+	+	+	?	?	–		9	Fair
**Swenson, et al. 2021**	+	+	+	+	–	+	+	+	+	+	+	?	?	–		10	Fair

Online resource 5 – Risk of bias assessment

**Wilcox, et al. 2015**	+	+	+	+	–	+	+	+	+	+	+	–	**?**	–		10	Fair

Criteria:

+ = Indicated a low risk of bias; ? = Indicates that there are some concerns; – = Indicates a high risk of bias.
