# Supplementary material for: Head Acceleration Magnitude in Sport-Related Concussive Impacts: A Systematic Review and Meta-analysis
Source: Sports Med. 2026 May 26;56(10):2477–95. doi: 10.1007/s40279-026-02448-x (PMC13614995; doi:10.1007/s40279-026-02448-x)
Supplement: Supplementary file 6 [file 40279_2026_2448_MOESM6_ESM.pdf]

Online resource 6 – Pairwise analyses

Head acceleration magnitude in sport-related concussive impacts:

A systematic review and meta-analysis

Branimir Ivanic[1], Anna Cronström[1], Mana Rasi[1], Eva Ageberg[1]

[1]Department of Health Sciences, Faculty of Medicine, Lund University, Lund, Sweden

Journal
Sports Medicine

Corresponding author
Branimir Ivanic, Department of Health Sciences, Faculty of Medicine, Lund University, Lund, Sweden
Email: branimir.ivanic@med.lu.se
Phone: +46 739 695 279

ORCID
Branimir Ivanic: 0009-0009-5144-6516
Anna Cronström: 0000-0002-3282-6320
Mana Rasi: 0009-0002-8599-6072
Eva Ageberg: 0000-0002-8639-3006

Online resource 6 – Pairwise analyses

Table 1. Pairwise mean difference in linear head acceleration: sport type.

	American Football	Ice hockey	Rugby	Soccer	Lacrosse	Australian Rules Football
**American Football**	–	+40.0g ($p < 0.001$)	+20.5g (p 0.14)	+31.4g (p 0.01)	+13.6g (p 0.33)	+14.8g (p 0.08)
**Ice hockey**		–	-19.5g (p 0.16)	-8.6g (p 0.47)	-26.4g (p 0.06)	-25.3g ($p < 0.001$)
**Rugby**			–	+10.9g (p 0.51)	-7.0g (p 0.70)	-5.8g (p 0.69)
**Soccer**				–	-17.8g (p 0.28)	-16.7g (p 0.18)
**Lacrosse**					–	+1.2g (p 0.93)
**Australian Rules Football**						–

Table 2. Pairwise mean difference in rotational head acceleration: sport type.

	American Football	Ice hockey	Rugby	Soccer	Lacrosse	Australian Rules Football
**American Football**	–	+635 rad/s^2 (p 0.40)	-3297 rad/s^2 (p 0.16)	-1203 rad/s^2 ($p < 0.001$)	-3255 rad/s^2 (p 0.06)	/
**Ice hockey**		–	-3932 rad/s^2 (p 0.10)	-1838 rad/s^2 ($p < 0.001$)	-3890 rad/s^2 (p 0.03)	/
**Rugby**			–	+2094 rad/s^2 (p 0.36)	+42 rad/s^2 (p 0.99)	/
**Soccer**				–	-2052 rad/s^2 (p 0.22)	/
**Lacrosse**					–	/
**Australian Rules Football**						–

Table 3. Pairwise mean difference in linear head acceleration: age group.

	Youth	High school	Collegiate	Adult
**Youth**	–	-27.0g ($p < 0.001$)	-14.2g (p 0.18)	-10.8g (p 0.12)
**High school**		–	+12.7 (p 0.25)	+16.2 (p 0.03)
**Collegiate**			–	+3.45 (p 0.74)
**Adult**				–

Table 4. Pairwise mean difference in rotational head acceleration: age group.

	Youth	High school	Collegiate	Adult
**Youth**	–	-1761 rad/s^2 (p 0.19)	-1092 rad/s^2 (p 0.23)	-3989 rad/s^2 (p 0.10)
**High school**		–	+669 rad/s^2 (p 0.60)	-2228 rad/s^2 (p 0.39)
**Collegiate**			–	-2897 rad/s^2 (p 0.22)
**Adult**				–

Online resource 6 – Pairwise analyses

Table 5. Pairwise mean difference in linear head acceleration: instrumentation type.

	**Helmets**	**Mouthguards**	**Skin patches**	**Headbands**
**Helmets**	–	+11.0g<br>(p 0.53)	-9.4g<br>(p 0.18)	+39.3g<br>($p < 0.001$)
**Mouthguards**		–	-1.6<br>(p 0.92)	+28.3<br>(p 0.14)
**Skin patches**			–	+29.9<br>($p < 0.001$)
**Headbands**				–

Table 6. Pairwise mean difference in rotational head acceleration: instrumentation type.

	**Helmets**	**Mouthguards**	**Skin patches**	**Headbands**
**Helmets**	–	-1709 rad/s^2<br>(p 0.33)	-2868 rad/s^2<br>(p 0.01)	/
**Mouthguards**		–	-1159 rad/s^2<br>(p 0.56)	/
**Skin patches**			–	/
**Headbands**				–
