# Supplementary material for: Head Acceleration Magnitude in Sport-Related Concussive Impacts: A Systematic Review and Meta-analysis
Source: Sports Med. 2026 May 26;56(10):2477–95. doi: 10.1007/s40279-026-02448-x (PMC13614995; doi:10.1007/s40279-026-02448-x)
Supplement: Supplementary file 7 [file 40279_2026_2448_MOESM7_ESM.pdf]

Online resource 7 – Sensitivity analyses

Head acceleration magnitude in sport-related concussive impacts: A systematic review and meta-analysis

Branimir Ivanic[1], Anna Cronström[1], Mana Rasi[1], Eva Ageberg[1]

[1]Department of Health Sciences, Faculty of Medicine, Lund University, Lund, Sweden

Journal
Sports Medicine

Corresponding author
Branimir Ivanic, Department of Health Sciences, Faculty of Medicine, Lund University, Lund, Sweden
Email: branimir.ivanic@med.lu.se
Phone: +46 739 695 279

ORCID
Branimir Ivanic: 0009-0009-5144-6516
Anna Cronström: 0000-0002-3282-6320
Mana Rasi: 0009-0002-8599-6072
Eva Ageberg: 0000-0002-8639-3006

Online resource 7 – Sensitivity analyses

Table 1. Sensitivity Analyses of SRC vs. Non-concussive Impacts.

Linear head acceleration				
	№ of studies	№ of head impacts	(SMD, 95% CI)	I^2 (%)
**Meta–analysis (all articles included)**	**15**	**175,952**	**2.42 (1.73 to 3.12)**	**87%**
Good/Fair articles only	10	75,101	1.94 (1.09 to 2.79)	79%
Video-verified impacts only	11	126,407	2.30 (1.50 to 3.09)	83%
≥10g threshold only	13	174,625	2.46 (1.74 to 3.17)	87%
Multiple SRC impacts only	8	163,013	2.73 (1.99 to 3.48)	89%
**Rotational head acceleration**				
	№ of studies	№ of participants	SMD (95% CI)	I^2 (%)
**Meta–analysis (all articles included)**	**9**	**401,996**	**1.99 (1.17 to 2.81)**	**89%**
Good/Fair articles only	7	366,765	2.00 (1.02 to 2.99)	90%
Video-verified impacts only	6	358,514	2.09 (0.88 to 3.29)	89%
≥10g threshold only	8	401,103	2.10 (1.24 to 2.96)	90%
Multiple SRC impacts only	5	394,066	2.01 (0.83 to 3.20)	95%
