# Supplementary material for: Head Acceleration Magnitude in Sport-Related Concussive Impacts: A Systematic Review and Meta-analysis
Source: Sports Med. 2026 May 26;56(10):2477–95. doi: 10.1007/s40279-026-02448-x (PMC13614995; doi:10.1007/s40279-026-02448-x)
Supplement: Supplementary file 8 [file 40279_2026_2448_MOESM8_ESM.pdf]

Online resource 8 – Reporting biases

Head acceleration magnitude in sport-related concussive impacts: A systematic review and meta-analysis

Branimir Ivanic[1], Anna Cronström[1], Mana Rasi[1], Eva Ageberg[1]

[1]Department of Health Sciences, Faculty of Medicine, Lund University, Lund, Sweden

Journal
Sports Medicine

Corresponding author
Branimir Ivanic, Department of Health Sciences, Faculty of Medicine, Lund University, Lund, Sweden
Email: branimir.ivanic@med.lu.se
Phone: +46 739 695 279

ORCID
Branimir Ivanic: 0009-0009-5144-6516
Anna Cronström: 0000-0002-3282-6320
Mana Rasi: 0009-0002-8599-6072
Eva Ageberg: 0000-0002-8639-3006

Online resource 8 – Reporting biases

Linear Head Acceleration (Egger's test: $p = 0.27$)

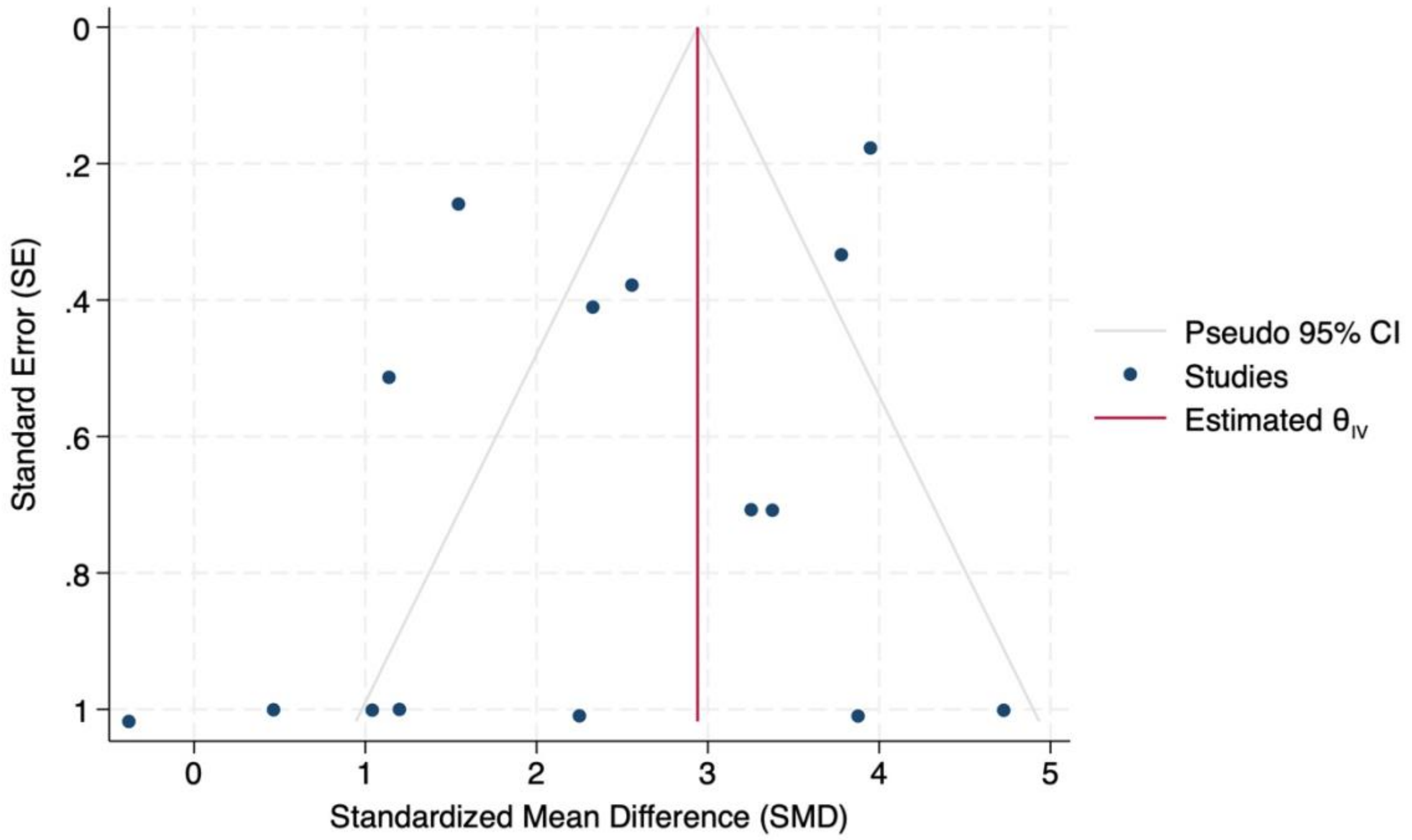

Figure 1. Funnel plot for assessing publication bias: Linear head acceleration.

Rotational Head Acceleration (Egger's test: $p = 0.41$)

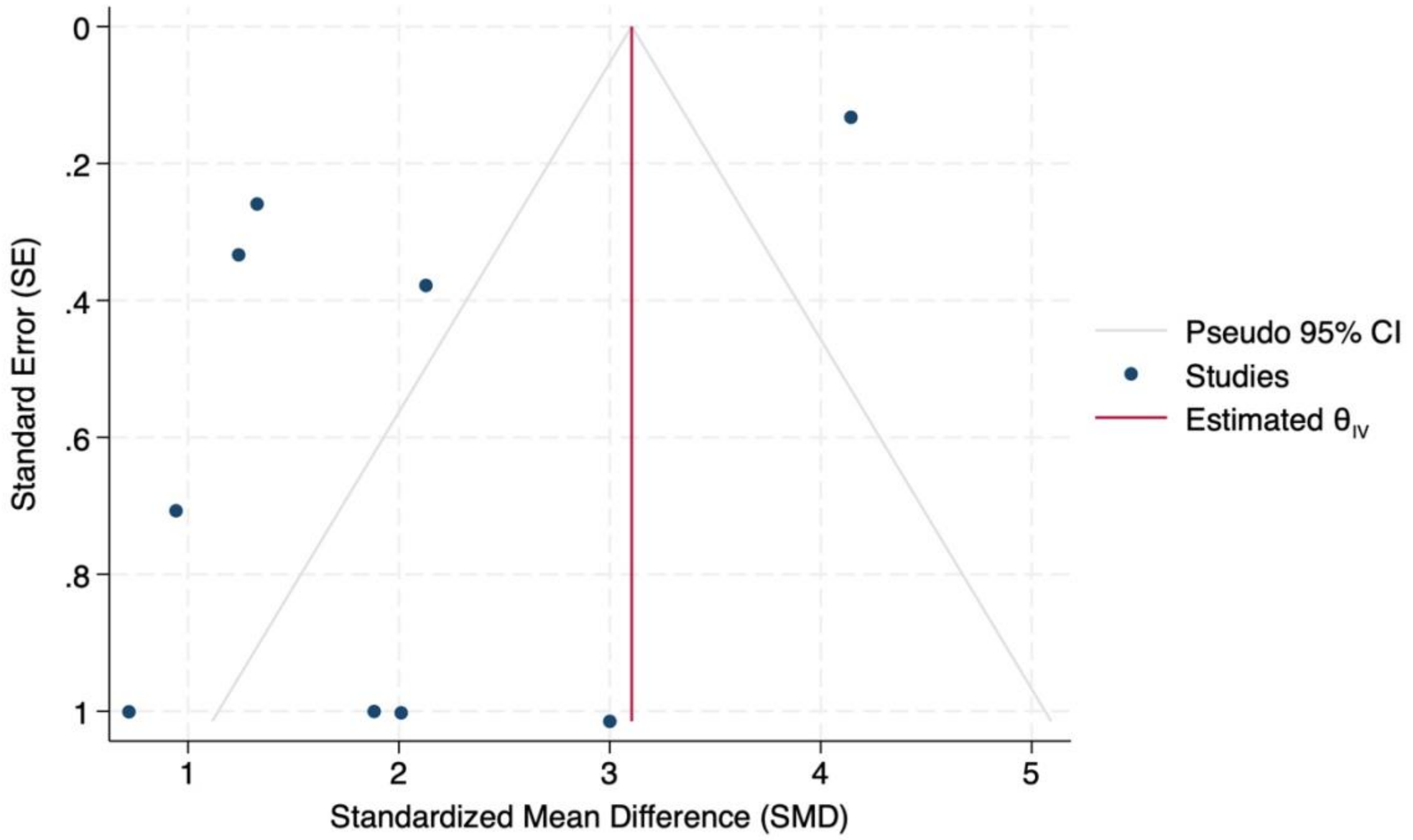

Figure 2. Funnel plot for assessing publication bias: Rotational head acceleration.
